# Lysophosphatidylcholine inhibits lung cancer cell proliferation by regulating fatty acid metabolism enzyme long‐chain acyl‐coenzyme A synthase 5

**DOI:** 10.1002/ctm2.1180

**Published:** 2023-01-13

**Authors:** Linlin Zhang, Xuanqi Liu, Yifei Liu, Furong Yan, Yiming Zeng, Yuanlin Song, Hao Fang, Dongli Song, Xiangdong Wang

**Affiliations:** ^1^ Department of Pulmonary and Critical Care Medicine Zhongshan Hospital, Fudan University Shanghai Medical College Shanghai China; ^2^ Shanghai Institute of Clinical Bioinformatics Shanghai China; ^3^ Center of Molecular Diagnosis and Therapy The Second Hospital of Fujian Medical University Quanzhou China; ^4^ Shanghai Engineering Research for AI Technology for Cardiopulmonary Diseases Shanghai China; ^5^ Department of Anesthesiology Zhongshan and Minhang Hospital Fudan University Shanghai China

**Keywords:** ACSL5, lipid metabolisms, lung cancer, LysoPC, transcriptomics

## Abstract

Lung cancer is a widespread malignancy with a high death rate and disorder of lipid metabolism. Lysophosphatidylcholine (lysoPC) has anti‐tumour effects, although the underlying mechanism is not entirely known. The purpose of this study aims at defining changes in lysoPC in lung cancer patients, the effects of lysoPC on lung cancer cells and molecular mechanisms. Lung cancer cell sensitivity to lysoPC was evaluated and decisive roles of long‐chain acyl‐coenzyme A synthase 5 (ACSL5) in lysoPC regulation were defined by comprehensively evaluating transcriptomic changes of ACSL5‐downregulated epithelia. ACSL5 over‐expressed in ciliated, club and Goblet cells in lung cancer patients, different from other lung diseases. LysoPC inhibited lung cancer cell proliferation, by inducing mitochondrial dysfunction, altering lipid metabolisms, increasing fatty acid oxidation and reprograming ACSL5/phosphoinositide 3‐kinase/extracellular signal‐regulated kinase‐regulated triacylglycerol‐lysoPC balance. Thus, this study provides a general new basis for the discovery of reprogramming metabolisms and metabolites as a new strategy of lung cancer precision medicine.

## Graphical abstract

1. LysoPC reprograms lipid metabolisms by increasing the accumulation of fatty acids and activating acyl‐CoA‐dominated metabolic mode under the control and regulation of ACSL5.
2. LysoPC up‐regulates the expression of ACSL5 and activates the ACSL5‐oriented lipid metabolism by promoting the phosphorylation of PI3K/mTOR and Ras/ERK signal pathways.
3. LysoPC inhibited lung cancer cell proliferation by promoting ACSL5, leading to the disorder of FA degradation.

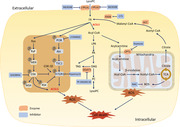

## 1 INTRODUCTION

Lung cancer is one of the leading causes of cancer‐related death, with approximately 2 million new cases and 17.76 million deaths each year.^1^ Carcinogenesis is attributed to the mutation of oncogenes, directly or indirectly affecting the metabolism of cancer cells.^2^ During the development of malignancy, cancer cells rewrite the metabolism or adapt unexpected metabolisms to maintain the energy needed for cell growth, division and survival, even in insufficient extracellular nutrients.^3^ Reprogrammed metabolisms secondarily control and decide cancer cell phenomes, capacities and progression.

Long‐chain acyl‐coenzyme A synthases (ACSLs) activate long‐chain fatty acids (FAs), contributing to lipid metabolisms, cell differentiation and death, organ function, inflammatory responses, carcinogenesis, cancer development and progression and patient prognosis.^4^ Of ACSLs, ACSL5 is nuclear‐coded, expressed in the mitochondria and involved in cell differentiation, maturation and death, by controlling the metabolism from long‐chain FA to fatty acyl‐CoA esters. Converting 12–20 chain length free long‐chain FA into fatty acyl‐CoA esters is the initial step in FA metabolism.^5^ Lipids (e.g. palmitic acid) regulate cell sensitivity, proliferation and intracellular energy by upregulating ACSL5 expression.^6^ ACSL5 modulates cell differentiation and proliferation by activating the pathway of Wnt2B palmitoylation, through which Wnt2B retains on mitochondrial membranes and participates in the pro‐apoptotic sensing of cells.^7^

ACSL5 maintains intracellular homeostasis and cancer cell death, malignance and recurrence by interacting with lipid metabolism‐related enzymes (e.g. acyl‐CoA dehydrogenase long chain, acyl‐CoA oxidases, carnitine palmitoyl transferase I [CPT1s], elongases of very long chain FA and fatty acid synthase [FASN]).^8^ The interaction of ACSL5 with non‐lipid metabolism‐associated proteins and ACSL5‐dominated protein‐protein networks regulates intracellular organelle communications, lipid metabolism and cancer cell proliferation, for example, interactions of ACSL5 with C‐C motif chemokine ligand 3,^6^ lipoxygenase,^8^ and TP53.^9^ Of those intracellular signal proteins, phosphoinositide 3‐kinase (PI3K) and isoforms control cell proliferation and death by regulating various metabolic pathways. PI3K family has 14 isoforms responsible for the phosphorylation of 3′ hydroxyl of the inositol ring of phosphatidylinositides to produce PI3K‐lipids and modulate the major stream of intracellular signals.^10^

Lysophosphatidylcholine (lysoPC) is derived from PCs by phospholipase A_2_, degraded to glycerophosphocholine and free FA (FFA) and catalyzed by lysophospholipase A1 and A2 extracellularly.^11^ The resulting FFAs can be taken up and incorporated into membrane phospholipids and neutral lipids. LysoPC is physiologically transported into cells through albumin or α‐1‐acid glycoprotein and converted to PCs by lysoPC acyltransferase in the presence of acyl‐CoA.^12^ LysoPC and its synthetic analogues are considered bioactive lysolipids and anti‐tumour lipids, with high capacities of cellular membrane penetration and domain re‐organization, broad interruption of intracellular signal transduction and secondary effects of diacylglycerol accumulation and in stabilization.^13^ LysoPC as a dominant lipid component of oxidized low‐density lipoprotein was found to inhibit melanoma cell adhesion and metastasis into the lung by altering tumour cell membrane morphology and impairing migratory ability.^14^ FFAs from lysoPC were integrated into cancer cells to increase membrane rigidity and inhibit cell invasion.^11^ Lower levels of lysoPC16:0 were found in patients with intrahepatic cholangiocarcinoma,^15^ ovarian cancer,^16^ colorectal cancer,^17^ prostate cancer,^18^ and lung cancer,^19^ correlated to tumour progression and recurrence, high‐risk postoperative complications and postoperative recurrence.^18^, ^20^ A reduction in lysoPC, an important intermediate in phosphatidylcholine biosynthesis and degradation, has been observed in patients with advanced cancer and metastasis, although the exact mechanisms remain unclear. LysoPC accumulated within liposomes and had strong antimetastatic effects through radical shifts in tumour cell membrane FA composition toward saturated status.^14^

Clinical lipidomics by integrating clinical phenomes and lipidomic profiles provides new insights into the understanding of lung cancer heterogeneity and lipid metabolism‐associated molecular mechanisms in lung cancer.^21^, ^22^ Clinical studies show that lipidomic profiles differentiate lung cancer stages, severity, subtypes and drug responses from non‐cancer lung diseases.^23^ Alterations of lipidomic profiles were suggested to apply for the identification and development of disease‐specific and phenome‐specific biomarkers and targets.^24^ Our previous studies demonstrated characters and patterns of circulating lipidomic profiles in patients with adenocarcinoma (ADC), squamous cell carcinomas (SCCs) or small cell lung cancer (SCLC) and defined heterogeneities of lipidomic characteristics among lung cancer subtypes, gender, ages, stages, metastatic status, nutritional status and clinical phenome severity.^19^ Plasma levels of lysoPC were obviously changed in ADC patients (5–12 folds), in < 60 years old patients (3–7 folds), at a late stage (2.5–3 folds), metastasis (2.5–3.5 folds), in patients with body mass index < 22 (2.5–4.5 folds) and in patients with digital evaluation score system scores < 90 (2.3–3.3 folds).^19^ However, little has been known about the direct effects of lysoPC on lung cancer cell proliferation and molecular mechanisms by which lysoPC changes the biological behaviours of lung cancer cells.

The aims of the current studies are to clarify changes in lysoPC in lung cancer patients, define the effects of lysoPC on lung cancer cells and explore the molecular mechanism. We performed clinical open random discovery and validation studies to confirm lung cancer subtype specificity of lipidomic profiles among subtypes and lung cancer specificity by comparing lipidomics of lung cancer with acute and chronic lung inflammations (e.g. acute pneumonia and chronic obstructive pulmonary disease [COPD]). We investigated the direct effects of exogenous lysoPC on lung cancer cells by screening lung cancer cell sensitivity, bio‐behaviours and proliferation. Potential roles of target gene ACSL5 in lysoPC regulation were uncovered by comprehensively evaluating transcriptomic changes of ACSL5‐down‐regulated epithelial and ACSL5 expression among single‐cell subtypes of lung epithelia among different lung diseases. We found that patients with acute pneumonia and COPD had higher plasma concentrations of total lysoPC than the healthy, especially increased 16:0, 16:1, 18:0 and 18:1 lysoPC species. External lysoPC activated the pathways of FA metabolism and increased the expression of the FA metabolic‐related gene ACSL5. External lysoPC inhibited lung cancer cell proliferation, mitochondrial dysfunction and lipid metabolisms by increasing FA oxidation through ACSL5, while down‐regulation of ACSL5 changed lung cancer cell sensitivity in response to lysoPC.

## 2 RESULTS

### 2.1 Levels and disease specificity of lysoPC in lung cancer patients

**FIGURE 1:**
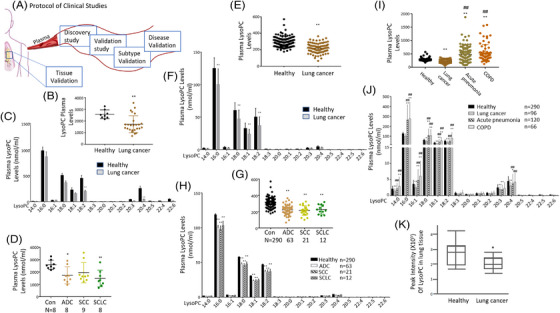
A series of clinical open random studies to evidence lysophosphatidylcholine (lysoPC) alterations. The protocol of clinical discovery and validation studies was designed prospectively (A), to investigate plasma levels of total lysoPC (B) and lysoPC 15 species (C) between lung cancer patients (*n* = 25) and healthy (*n* = 8) as well as plasma levels of total lysoPC (D) in lung cancer subtypes (adenocarcinoma [ADC] [*n* = 8], squamous cell carcinoma [SCC] [*n* = 9] or small cell lung cancer [SCLC] [*n* = 8]) in the discovery study. The validation studies included plasma levels of total lysoPC (E) and lysoPC species (F) between healthy (*n* = 290) and lung cancer patients (*n* = 96) as the first part; plasma levels of total lysoPC (G) and lysoPC species (H) between healthy and patients with ADC (*n* = 63), SCC (*n* = 21) and SCLC (*n* = 12) as the second part; plasma levels of total lysoPC (I) and lysoPC species (J) between healthy and patients with lung cancer, acute pneumonia (*n* = 120) or COPD (n = 66) as the third part, as well as lung tissue lysoPC levels (K) between lung cancer tissues and corresponding para‐cancer tissues (*n* = 10 pairs). * and ** stand for the *p*‐values less than .05 and .01, respectively, as compared with the healthy

We first evaluated the importance of lysoPC in lung cancer patients as shown in the protocol of clinical studies (Figure 1A), performed a discovery study to measure plasma levels of lipidomic profiles in 25 patients with lung cancer and mapped the different patterns between healthy and lung cancers (Figure 1B,C). As compared with healthy, lipid elements increased or decreased significantly in lung cancer patients, as shown in Table S1 (*p* < .05). Of those elements, levels of lysoPC elements in lung cancer were significantly lower than those in the healthy (Figure 1B, *p* < .01). Of declined lysoPC elements (*n* = 15), levels of lysoPC (18:2) and (20:4) showed statistically different (Figure 1C). ADC and SCLC had significantly lower levels of lysoPCs (Figure 1D, *p* < .05). To validate the preliminary results from the discovery study, we measured lysoPC elements in a large population of healthy (*n* = 290) and patients with lung cancer (*n* = 96) and found that the levels of lysoPCs in lung cancer patients were markedly lower compared to the healthy (Figure 1E, *p* < .01). Of 16 lysoPCs, levels of lysoPCs 16:0, 18:0, 18:1 and 18:2 in patients with lung cancer were significantly lower (Figure 1F, *p* < .01).

We evaluated the specificity of lung cancer subtypes and found a significant decline in ADC and SCC (Figure 1G, *p* < .01). Of declined lysoPCs elements, there was no difference among lung cancer subtypes (Figure 1H). To evaluate the disease specificity of lysoPCs, we compared plasma levels of lysoPCs between patients with lung cancer or with non‐cancer lung diseases, for example, acute pneumonia and COPD and found that levels of lysoPCs in acute pneumonia and COPD were significantly increased compared with healthy and lung cancers (Figure 1I, *p* < .01). Of lysoPC elements, levels of lysoPCs 16:0, 18:0, 18:1 and 18:2 were significantly elevated in acute pneumonia and COPD when compared with healthy and patients with lung cancer (Figure 1J, *p* < .05), of which increased or decreased lysoPCs were detailed and listed in Table S2. To validate the compatibility of lysoPCs between circulation and lung tissue, we measured tissue levels of lysoPCs in lung cancer and corresponding normal tissues and found significantly lower levels in lung cancer homogenates (Figure 1K, *p* < .05).

### 2.2 Inhibitory roles of exogenous lysoPCs in lung cancer cells

**FIGURE 2:**
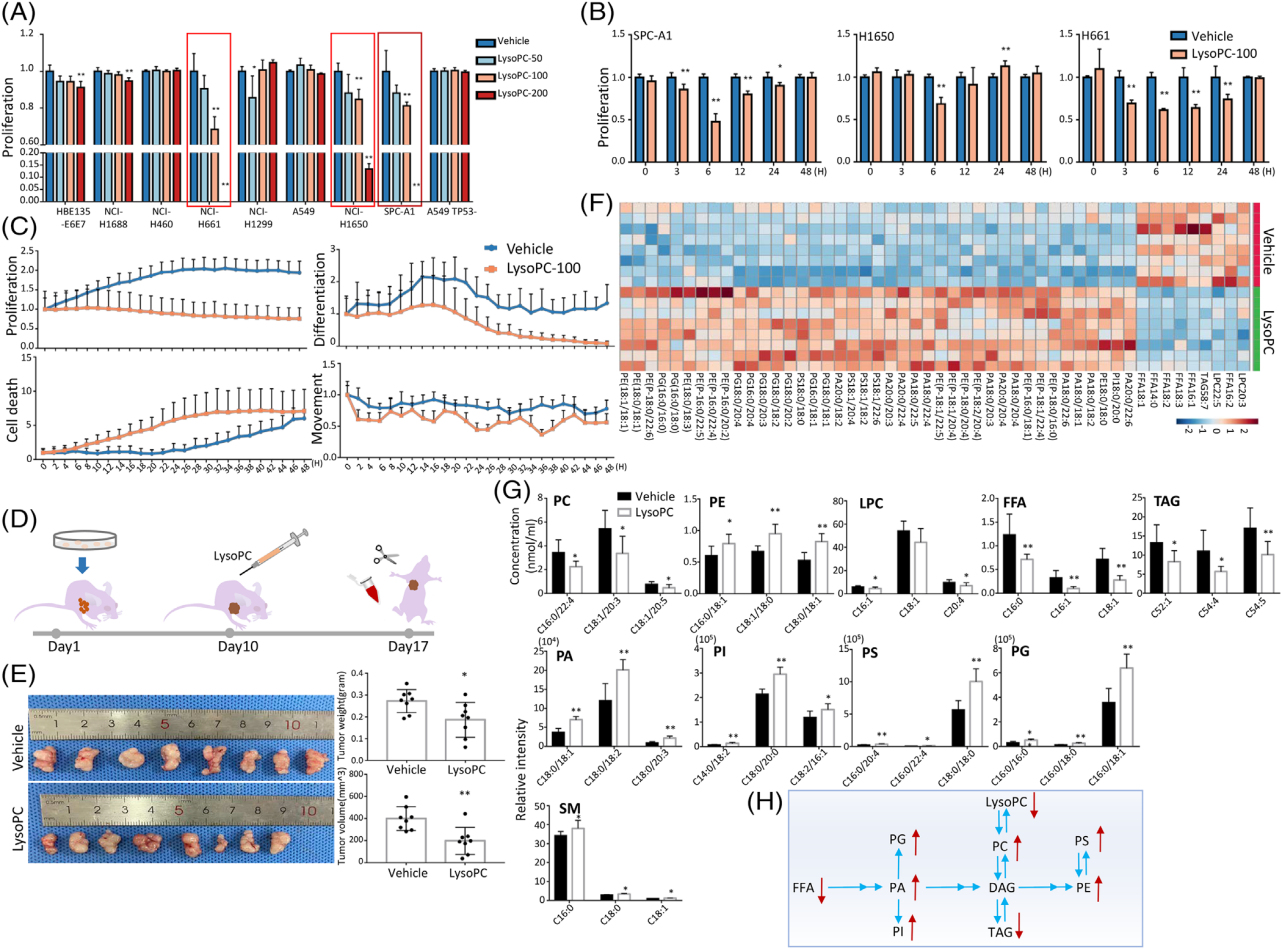
Validation of exogenous lysophosphatidylcholine (lysoPC) effects on lung cancer in preclinical studies, Effects of exogenous lysoPC at different concentrations on the proliferation of nine lung epithelial cells (A; *n* = 5 per group) was validated, from which the proliferation of SPC‐A1, NCI‐H1650 and NCI‐H661 was assessed after once treatment with lysoPC at the concentration of 100 μM for time points (B) and bio‐behaviours (proliferation, death, differentiation and movement) of SPC‐A1 cells after continuous treatment with lysoPC at 100 μM for 48 h (C; *n* = 12 per group). The protocol of evaluation on the effects of lysoPC in an animal model was shown in D. Human SPC‐A1 cells were transplanted into the armpits of mice on day 1 and the tumours grew for 10 days. The experimental group was intraperitoneally injected with vehicle or lysoPC on day 10 and the experiment was terminated seven consecutive days after the injection, and the tumour and blood were sampled for further analyses, including mouse tumour weight and size (E), heatmap of top 50 lipid levels with statistical significance (F) and plasma levels of representative lipid species (G) between animals treated with vehicle or lysoPC (*n* = 8 per group). The metabolic pathway of lipid changes in the blood of an in vivo mouse model was also described (H). * and ** stand for the *p*‐values less than .05 and .01, respectively, as compared with the groups treated with vehicle

To explore the effects of lysoPC on lung cancer cells, we screened the sensitivity of lung epithelial cells (*n* = 9) to lysoPC at different concentrations and found that NCI‐H661, NCI‐H1650 and SPC‐A1 cells appeared dose‐dependent sensitivities (Figure 2A). SPC‐A1 and NCI‐H661 began to respond to exogenous lysoPCs at 100 μM between 3 and 48 h (Figure 2B). During the exposure to lysoPC, SPC‐A1 showed dynamic responses to lysoPC in Figure 2C, where the dynamic rates of SPC‐A1 proliferation and differentiation continuously decreased and the number of dead cells increased during 48 h after exposure to lysoPC (Figure 2C). We constructed an SPC‐A1‐derived xenograft nude mouse model and observed tumour growth changes after intraperitoneal injection of lysoPC (Figure 2D). The lysoPC‐treated animals had slower tumour growth and lower tumour size and weight than vehicle‐treated ones (Figure 2E). We analyzed plasma lipidomic profiles of mice and found the top 50 lipids with statistical differences between animals with or without lysoPC supplement (Figure 2F). Of those, PC, PE, PA, PI, PS, PG and SM increased, while lysoPC, triacylglycerol (TAG) and FFA decreased (Figure 2G). The results suggested an increase in FFA consumption and a shift in the metabolism of various phospholipids (Figure 2H).

### 2.3 Specificity of ACSL5‐expressed in lung cell‐line and lung epithelia among lung diseases

**FIGURE 3:**
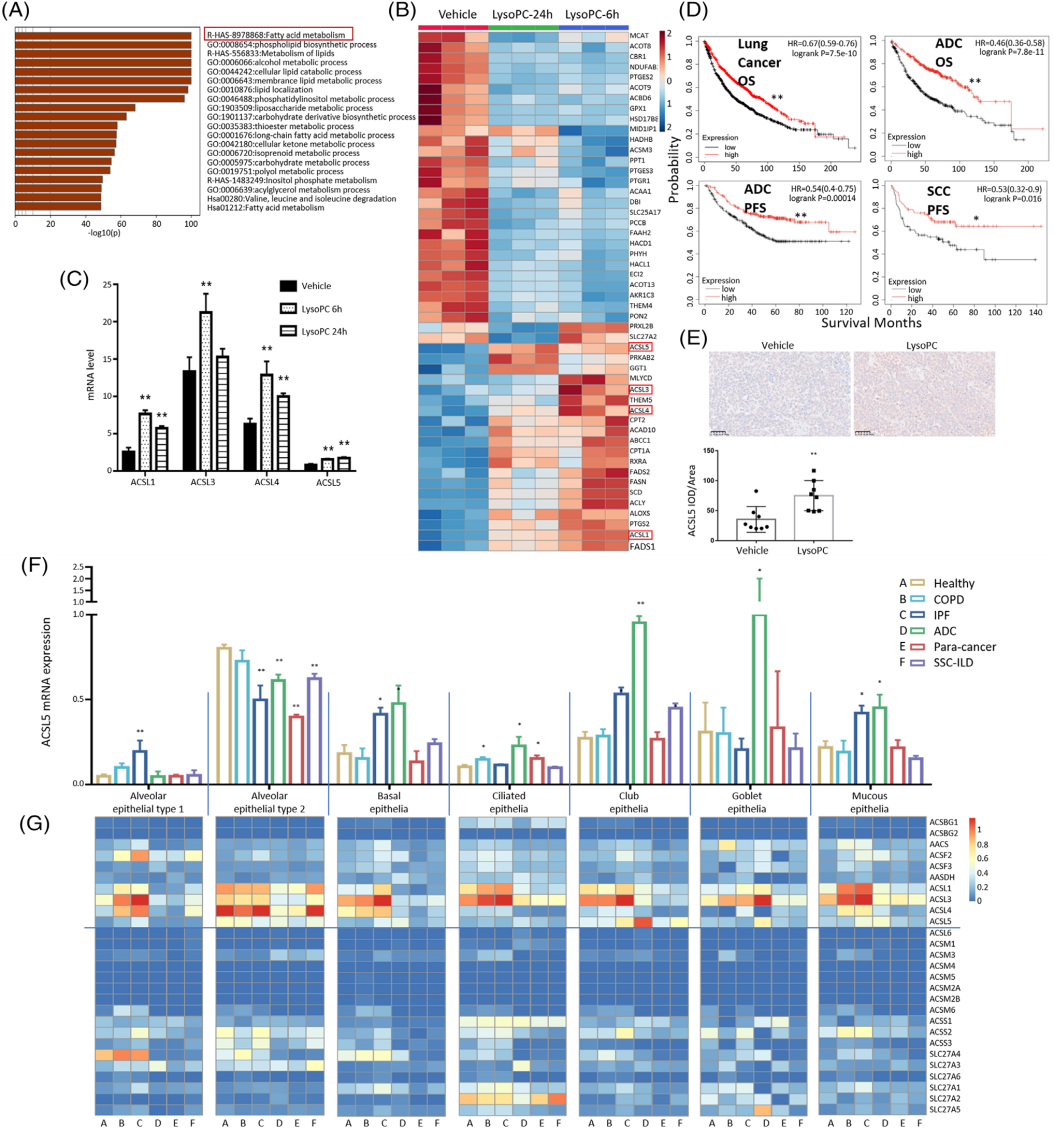
Screening, selection and validation of target gene long‐chain acyl‐coenzyme A synthases 5 (ACSL5) in lysophosphatidylcholine (lysoPC) effects. (A) The lysoPC‐specific genes and pathways using RNA‐seq were screened and selected in cells treated with vehicle or lysoPC for 24 h, by matching human lipid‐related genes for pathway enrichment (https://metascape.org/). Of the top 50 differently expressed genes related to fatty acid metabolism (B), ACSL family members, for example, ACSL1, ACSL3, ACSL4 and ACSL5 over‐expressed 6 and 24 h after treatment with lysoPC and presented digitally in (C) (*n* = 3 per time point and *n* = 6 per group). Of the ACSL family, the overall survival rate (OS) and progression‐free survival rate (PFS) of lung cancer or adenocarcinoma (ADC) patients with ACSL5 high‐ and low‐expression were validated using Kaplan‐Meier Plotter of survival data (D). Spatial expression of ACSL5 in preclinical lung cancer tissues was measured by intra‐tumoural immunohistochemistry detection of the ACSL5 gene in mice model (E). The expression (F; *n* = 27) and heatmap (G) of ACS family member genes were validated in lung single epithelial cells from lung tissues of normal (*n* = 20), chronic obstructive pulmonary diseases (COPD; *n* = 15), idiopathic pulmonary fibrosis (IPF; *n* = 15), lung adenocarcinoma (ADC; *n* = 15), systemic sclerosis‐associated interstitial lung disease (SSC; *n* = 8) or para‐cancerous tissue (para‐cancer; *n* = 11) by deep‐mining of single‐cell RNA‐seq datasets (GSE128169, GSE131907 and GSE136831). * and ** stand for the *p*‐values less than .05 and .01, respectively, as compared with the groups treated with vehicle or healthy

The effects of lysoPCs on transcriptomic profiles of SPC‐A1 cells were assessed 6 and 24 h after exposure to lysoPCs at 100 μM. FA metabolism and associated pathways were enriched on basis of functional genes among signal pathways with statistical significance, as compared to cells treated with vehicle (Figure 3A, *p* < .05). The differentially expressed genes (DEGs) were matched with FA‐related genes and correlated with corresponding proteins and protein‐protein interaction networks (Figure S1A). Of the top 50 FA‐related DEGs measured by RNA‐seq and shown in the heat map (Figure 3B), the ACSL family up‐regulated in lysoPCs‐treated cells, especially at 6 h, as compared with vehicle‐treated cells. We found that ACSL family members were the centre of DEGs networks and interactions (Figure S1B). The mRNA expression of ACSL1 and ACSL4 significantly increased in SPC‐A1 cells at 6 and 24 h after lysoPC treatment, ACSL3 at 6 h and ACSL5 continuously in a time‐dependent manner (Figure 3C, *p* < .01) and a dose‐associated pattern (Figure S1C). Of ACSL family members, ACSL5 is highly expressed in SPC‐A1, NCI‐H1650 and NCI‐H1688 cells without any treatment as compared with other cells (Figure S1D).

We analyzed the potential influences of the selected target gene ACSL5 in patient survival and found that ACSL5‐high‐expressing patients in lung cancer tissues and ADC tissues had significantly better 10‐year overall survival rates than that of the patient with low expression (Figure 3D, *p* < .01, respectively). The progression‐free survival rate was higher in ADC and SCC tissues of patients with ACSL5 high expression (Figure 3D). The numbers at risk in overall survival and progression‐free survival of patients with lung cancer, ADC and SCC during 10–15 years between high‐ or low‐expression of ACSL5 were listed in Figure S1E. The expression of ACSL5 proteins by immunohistochemical staining was higher in ADC tissues than in the adjacent tissues (Figure S1F). The expression of ACSL5 mRNA was also measured in HBE135‐E6E7, A549 ^p53+^, A549 ^p53−^, NCI‐H1650, NCI‐H1688, NCI‐H1299, NCI‐H460 and NCI‐H226 cells 24 h after treatment with lysoPC at concentrations of 25, 50 and 100 μM (Figure S1G). We noted that mRNA expression of ACSL5 down‐regulated in HBE, A549*^p53+^* and NCI‐H1650 cells, while up‐regulated in A549*^p53−^*, NCI‐H1688, NCI‐H1299 and NCI‐H460 after being treated with lysoPC at high concentrations. After the procedures of screening and validating (Figure S2A), ACSL5 was chosen as the key gene and SPC‐A1 was the target cell for further investigation. The in vivo experiment demonstrated that the expression of ACSL5 protein was elevated in tumours of mice treated with lysoPC (Figure 3E).

To define the expression of ACSL5 in human lung epithelia, we evaluated mRNA expressions of acyl‐coenzyme A synthase (ACS) family members (*n* = 27) in alveolar epithelial type I (ATI) and II (ATII), airway basal, ciliated, club, Goblet and mucous epithelia by mining scRNA‐seq databases. Expression of ACSL5 mRNA in ATII was higher than other epithelial types of healthy, ATI of idiopathic pulmonary fibrosis (IPF), basal epithelia of IPF and ADC, ciliated epithelia of COPD, ADC and para‐cancer tissues, club epithelia of IPF, ADC and systemic sclerosis‐associated interstitial lung disease (SSC), Goblet epithelia of ADC and mucous epithelial of IPF and ADC (Figure 3F), as compared with those in corresponding epithelia of healthy. ACSL5 expression is down‐regulated in ATII of IPF, ADC, para‐cancer and SSC tissues. Of ASC family members (Figure 3G), expression of ACSL1 mRNA up‐regulated in mucous epithelia of COPD and IPF, ACSL3 in ATI of COPD, basal epithelia of IPF, ciliated epithelia of COPD and IPF, club epithelia of IPF, Goblet cells of ADC, mucous epithelia of COPD and IPF and ACSL4 in ATI and ATII of IPF. Statistical analyses demonstrated that mRNA expression of ACSL1 (Figure S2B), ACSL3 (Figure S2C) and ACSL4 (Figure S2D) in lung and airway epithelial cells among lung diseases. As compared with ACSL subfamily members, ACSL5 mRNA is mainly over‐expressed in airway epithelial cells of patients with ADC. It indicates that ACSL5 may participate in epithelial proliferations and hyperplasia (basal cells), increased clearance (ciliated cells), constructive framing and supports (club cells) and mucous hyperproduction (Goblet and mucous cells) in the pathogenesis of ADC.

### 2.4 Regulatory roles of ACSL5 in regulating genes involved in lipid metabolism

**FIGURE 4:**
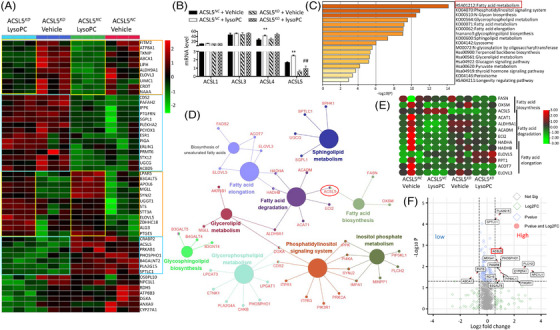
Validation of long‐chain acyl‐coenzyme A synthases 5 (ACSL5)‐dependent roles after down‐regulation of ACSL5. Transcriptomic profiles of ACSL5*^NC^* and ACSL5*^KD^* with vehicle or lysophosphatidylcholine (lysoPC) were measured by bulk RNA‐seq, of which the heatmap of top 50 lipid‐associated differentially expressed genes (A), the exact levels of ACSL family member genes (B) and the signal pathway enrichment of those genes (C) were presented (*n* = 3 per group and repeated thrice). ACSL5‐dominated lipid metabolism gene networks using Cytoscape (D), heatmap of genes involved in fatty acid biosynthesis, degradation and elongation (E) and volcano plot of lipid‐related genes (F) between ACSL5*^NC^* and ACSL5*^KD^* cells treated with vehicle or lysoPC were calculated, on basis of up‐regulated genes with fold changes > 1.5 and down‐regulated genes with fold changes < 0.67 (*p* < .05). * and ** or # and ## stand for the *p*‐values less than .05 and .01, respectively, as compared with ACSL5*^NC^* cells treated with vehicle or lysoPC. FASN: fatty acid synthase; OXSM: 3‐Oxoacyl‐ACP Synthase, mitochondrial; ACSL5: acyl‐CoA synthetase long‐chain family member 5; ACAT1: acetyl‐CoA acetyltransferase 1; ALDH9A1: aldehyde dehydrogenase 9 family member A1; ACADM: acyl‐CoA dehydrogenase medium chain; ECI2: enoyl‐CoA delta isomerase 2; HADHA: hydroxyacyl‐CoA dehydrogenase trifunctional multienzyme complex subunit alpha; HADHB: hydroxyacyl‐CoA dehydrogenase trifunctional multienzyme complex subunit beta; ELOVL4: ELOVL fatty acid elongase 4; PPT1: palmitoyl‐protein thioesterase 1; ACOT7: acyl‐CoA thioesterase 7; and ELOVL3: ELOVL fatty acid elongase 3

To explore the role of the ACSL5 gene in the regulation of genes associated with lipid metabolism, transcriptomic profiles were assessed in SPC‐A1 cells with knockdown of ACSL5 by small interference RNA (ACSL5*^KD^*) or with non‐specific sequences as negative control (ACSL5*^NC^*) after treated with vehicle or lysoPC at 100 μM for 24 h. Up‐ or down‐regulated expressions of transcriptomic genes were detailed in Table S3. Of the top 50 lipid metabolism‐associated DEGs between ACSL5*^KD^* and ACSL5*^NC^*, we found that a panel of genes was ACSL5‐dependent and only up/down‐regulated in ACSL5*^KD^* cells, as compared with those in ACSL5*^NC^* or a panel of lysoPC‐responding genes up/down‐regulated in lysoPC‐treated cells, as compared with vehicle‐treated cells (Figure 4A). Table S4 represents the significant difference of those genes between ACSL5*^KD^* and ACSL5*^NC^*, between ACSL5*^KD^* with lysoPC and vehicle and between ACSL5*^KD^* and ACSL5*^NC^* with lysoPC (p < 0.05). Of ACSL family members, expression of ACSL4 and ACSL5 mRNA up‐regulated in lysoPC‐treated cells, rather than in ACSL5*^KD^* cells (Figure 4B), illustrating the quality of target gene knockdown and less sensitivity. Most DEGs were associated with FA metabolism, phosphatidylinositol signalling system and glycan biosynthesis, as shown in Figure 4C. Among those signal pathways and functions, FA degradation was directly linked with FA biosynthesis by ACSL5, with FA elongation by HADHA and HADHB and with glycerolipid metabolism by ALDH9A1 (Figure 4D). LysoPC‐induced hyper/hypo‐expressions of FA metabolism‐associated genes were changed in ACSL5*^KD^* cells (Figure 4E). Among top DEGs, FASN, OXSM and ACSL5 were related to FA formation, ACSL5, ACAT1, ALDH9A1, ACADM, ECI2, HADHA and HADHB to FA degradation and HADHA, HADHB, ELOVL5, PPT1, ACOT7 and ELOVL3 to FA elongation. The majority of lipid metabolism‐associated DEGs were more than 1.5 folds and were related to FA metabolism between ACSL5*^KD^* cells with vehicle or lysoPC (Figure 4F).

### 2.5 LysoPC induced mitochondrial dysfunction and altered lipid metabolism

To investigate the direct effects of exogenous lysoPC on lung cancer cell mitochondria and lipid metabolisms, we measured mitochondrial morphology and function, cell death and lipid metabolisms of SPC‐A1 cells after lysoPC treats. Exogenous lysoPC‐induced changes in mitochondrial shape, fragmented and rounded phenomes, swelling and destruction of the mitochondrial ridges and mitochondrial vacuolization were observed under electric microscopy (Figure S3A), as compared with vehicle‐treated cells. The ratio of mitochondrial length/width significantly reduced in lysoPC‐treated SPC‐A1 (Figure S3B, *p* < .01), accompanied by alterations of mitochondrial structure and distribution by fluorescein staining (Figure S3C) and compromises of mitochondrial ridges and membrane function by reduction of mitochondrial texture density and mitochondrial membrane potential (Figure S3D). We evaluated the effects of lysoPC on mitochondria‐associated FA metabolism by measuring the rate‐limiting enzyme, CPT1, for the translocation of FA from the cytosol to the mitochondrial matrix and found that the amount of CPT1 proteins increased and distributed from perinuclear to intracellular diffusion and peri‐membrane (Figure S3E). Fatty acid oxidation mRNA expression of CPT1 subtype genes (CPT1A, CPT1B and CPT1C) up‐regulated in lysoPC‐treated cells, as compared with vehicle‐treated cells (Figure S3F). To assess the function of mitochondria‐interacted organelles, we measured endoplasmic reticulum stress markers, G protein‐coupled receptor 78 (GRP78) and X‐Box binding protein 1,^25^ and found that lysoPC induced over‐expression of both mRNAs (Figure S3G). GSK2656157 and 4μ8C are inhibitors of PRKR‐like endoplasmic reticulum (ER) kinase and inositol‐requiring enzyme 1 alpha and two key upstream players in ER stress cascades. To define whether elevated ER stress leads to LysoPC‐induced cell death, we used GSK2656157 and 4μ8C to inhibit ER stress and found that these inhibitions failed to rescue cell death induced by lysoPC (Figure S3H and S3I).

Exogenous lysoPC increased the production of reactive oxygen species (ROS) in a dose‐dependent pattern (Figure S3J), which was inhibited by the addition of antioxidant N‐Acetyl‐L‐cysteine (NAC) at 5 mM for 24 h (Figure S3K). NAC treatment could prevent lysoPC‐induced epithelial cell death (Figure S3L). The administration of etomoxir (100 nM, 24 h), an inhibitor of CPT1, inhibited lysoPC‐induced cell proliferation and reduces ROS production (Figure S3M,N). Exogenous lysoPC significantly increased intracellular levels of DAG, TAG, PE, PC, lysoPC, PG and AC, while the majority of PI, PS and SM species decreased (Figure S4A, *p* < .05). PA species C18:0 and its associates decreased, while C20:0 and its associates increased. It seems that exogenous lysoPC may activate the pathway of metabolism mainly from acyl‐CoA to PG through PA and to lysoPC, TAG and PE through DAG or DAG and PC, while deactivating the path to PI, SM and PS, as shown in Figure S4B. The expression levels of mRNAs involved in fatty acid oxidation (e.g. PPARA, SLC25A20 and ACADM) and lipogenesis (e.g. SREBF1, FASN and ACACA) significantly increased after lysoPC treatment (Figure S4C,D, *p* < .01). The expression of genes related to fatty acid uptake (e.g. CD36, SCL27A2 and SLC27A5) or mitochondrial biogenesis‐related genes increased or decreased, respectively (Figure S4E,F).

To evaluate the side effects and toxicity of external lysoPC, the volume (100 μl) of lysoPC at 40 mg/kg/day was intraperitoneally given into mice for 7 days and changes in mouse body weight were monitored every day. The pathological scores of the lung, liver and brain did not show a significantly different between mice with lysoPC or vehicle at the same volume (Figure S5A). There were no discernible changes in mouse body weight (Figure S5B). Lipid analysis revealed no significant difference between mice treated with lysoPC or vehicle (Figure S5C). On the other hand, we also observed the role of ACSL5 in the lysoPC effect in vivo. Although the effect was not statistically significant, we observed a trend toward inhibition of tumour growth by lysoPC. Furthermore, the ACSL5*^KD^* group decreased this suppression effect of lysoPC (Figure S5D). There was no difference in mouse body weights among mice with or without lysoPC (Figure S5E).

### 2.6 Regulatory roles of ACSL5 in exogenous lysoPC effects

**FIGURE 5:**
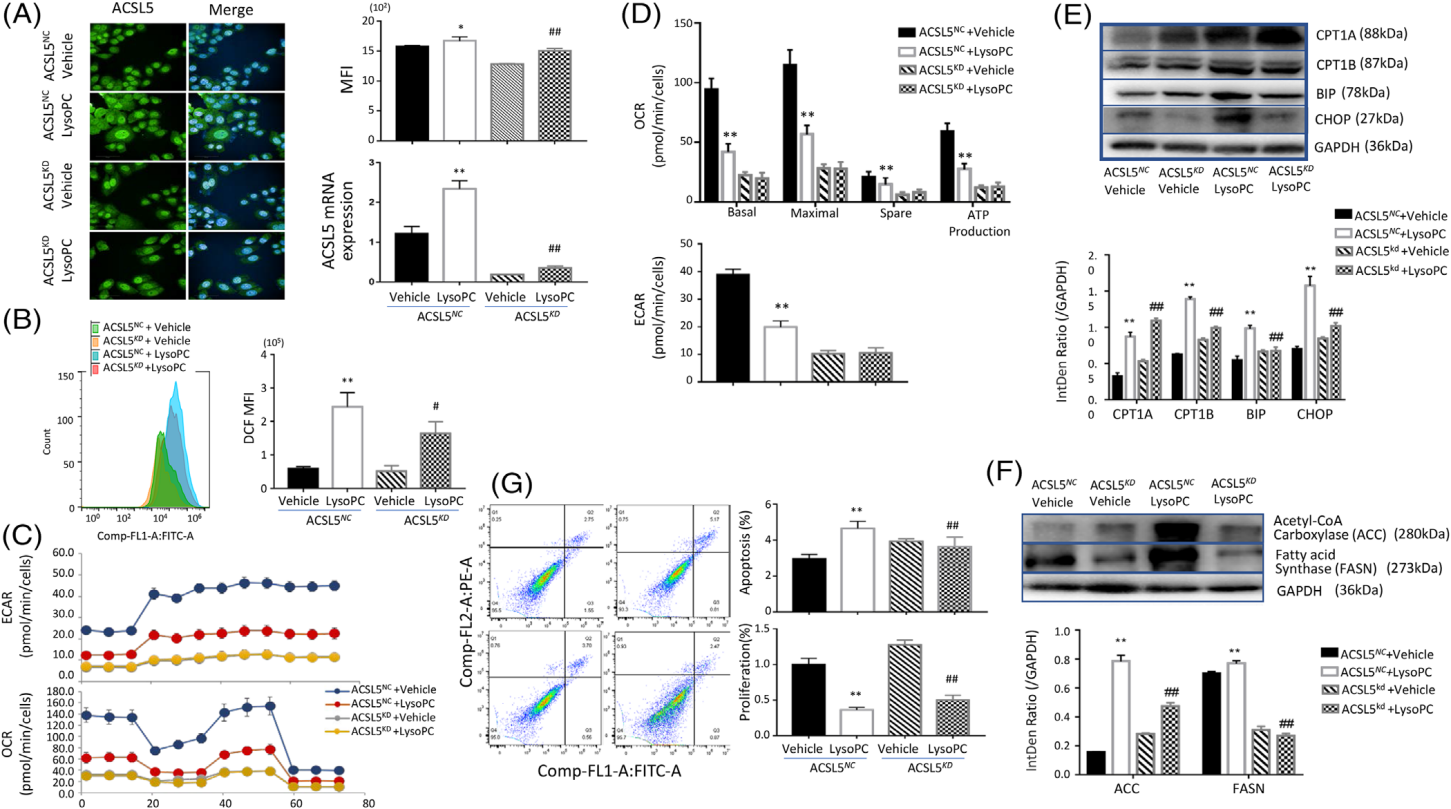
Lysophosphatidylcholine (lysoPC)‐induced mitochondrial dysfunctions through long‐chain acyl‐coenzyme A synthases 5 (ACSL5). LysoPC‐induced alterations of ACSL5 mRNA and protein expression were confirmed in ACSL5*^NC^* and ACSL5*^KD^* cells treated with vehicle or lysoPC for 6 h (A; representative fluorescence image with scale bar at 50 μm, *n* = 4 per group). Levels of reactive oxygen species (ROS) production (B), oxygen consumption rate (OCR) (C) and extracellular acidification rate (ECAR) (D) were measured to show critical roles ACSL5 in lysoPC‐changed mitochondrial function (n = 3 per group thrice). Protein levels of CPT1A, CPT1B, BIP and CHOP (F) as well as ACC and FASN (E) were assessed to detect lysoPC‐induced fatty acid hyper‐oxidations and ER dysfunction. Cell apoptosis and proliferation were evaluated (G) to evidence the central role of ACSL5 in inhibitory effects of lysoPC on lung cancer cells). * and ** or # and ## stand for the *p*‐values less than .05 and .01, respectively, as compared with of ACSL5*^NC^* cells treated with vehicle or lysoPC. CPT1: carnitine palmitoyl transferase I; BIP: binding‐immunoglobulin protein; CHOP: C/EBP‐homologous protein; and GAPDH: glyceraldehyde‐3‐phosphate dehydrogenase

We evaluated how ACSL5 participates in the process of exogenous lysoPC effects on mitochondrial and ER functions and metabolisms and found that lysoPC significantly increased the expression of ACSL5 mRNA and proteins in ACSL5*^NC^*, but not in ACSL5*^KD^* after being treated with vehicles (Figure 5A, *p* < .05). We noticed that levels of ROS production induced by lysoPC in ACSL5*^KD^* were significantly lower than in ACSL5*^NC^* (Figure 5B, *p* < .05). We overexpressed the ACSL5 gene in SPC‐A1 cells by plasmid (ACSL5*^OE^*), verified the effect of up‐regulation of ACSL5 gene on cell proliferation and ROS and evidenced the over‐expression of ACSL5 mRNA and protein after the selection and validation (Figure S6A,B). ACSL5*^OE^* accelerated the cell sensitivity and inhibitory ability of exogenous lysoPC on cell proliferation and induced more ROS production (Figure S6C,D). LysoPC reduced oxygen consumption rate (OCR) and extracellular acidification rate (ECAR), mitochondrial respiratory capacities, adenosine triphosphate production and ECAR in ACSL5*^NC^* 6 h after lysoPC administration, rather than in ACSL5*^KD^* (Figure 5C,D).

We evaluated the potential roles of ACSL5 in lysoPC‐induced fatty acid hyper‐oxidations and ER dysfunction and found that protein expression of CPT1A, CPT1B, ER heat shock protein 70 family member (BIP) and CCAAT/enhancer‐binding proteins homologous protein (CHOP), two important biomarkers and players of ER stress,^26^, ^27^ highly expressed in ACSL5*^NC^* cells treated with lysoPCs, as compared with cells with vehicle or ACSL5*^KD^* cells with lysoPC (Figure 5E), except for CPT1A. Levels of acetyl‐CoA carboxylase and FASN proteins in ACSL5*^NC^* cells were significantly higher than those in ACSL5*^KD^* cells in response to lysoPC and levels of ACC or FASN in ACSL5*^KD^* cells with lysoPC were higher or lower than ACSL5*^KD^* with a vehicle, respectively (Figure 5F, *p* < .01). The rates of cell apoptosis or proliferation in ACSL5*^KD^* cells were significantly lower or higher than in ACSL5*^NC^* cells after lysoPC treats (Figure 5G, *p* < .01). To examine the potential feed‐back regulation of ACSL5 in other ACSL family members, we measured the expression of ACSL family member mRNA in ACSL5*^KD^* cells and effects of other ACSL member down‐regulation on lysoPC‐induced cell death. Our results showed that ACSL5*^KD^* increased ACSL4 gene expression (Figure S6E). We developed ACSL4*^KD^* cells after screening and selection and examined changes in lysoPC‐induced cell proliferation in ACSL4*^KD^* cells. We found that ACSL4*^KD^* increased lysoPC‐induced cell death, opposite of ACSL5*^KD^* (Figure S6F) and failed to increase the gene expression of ACSL5 (Figure S6G).

### 2.7 Roles of ACSL5 and PI3K signal pathways in lysoPC‐induced lipid metabolisms

**FIGURE 6:**
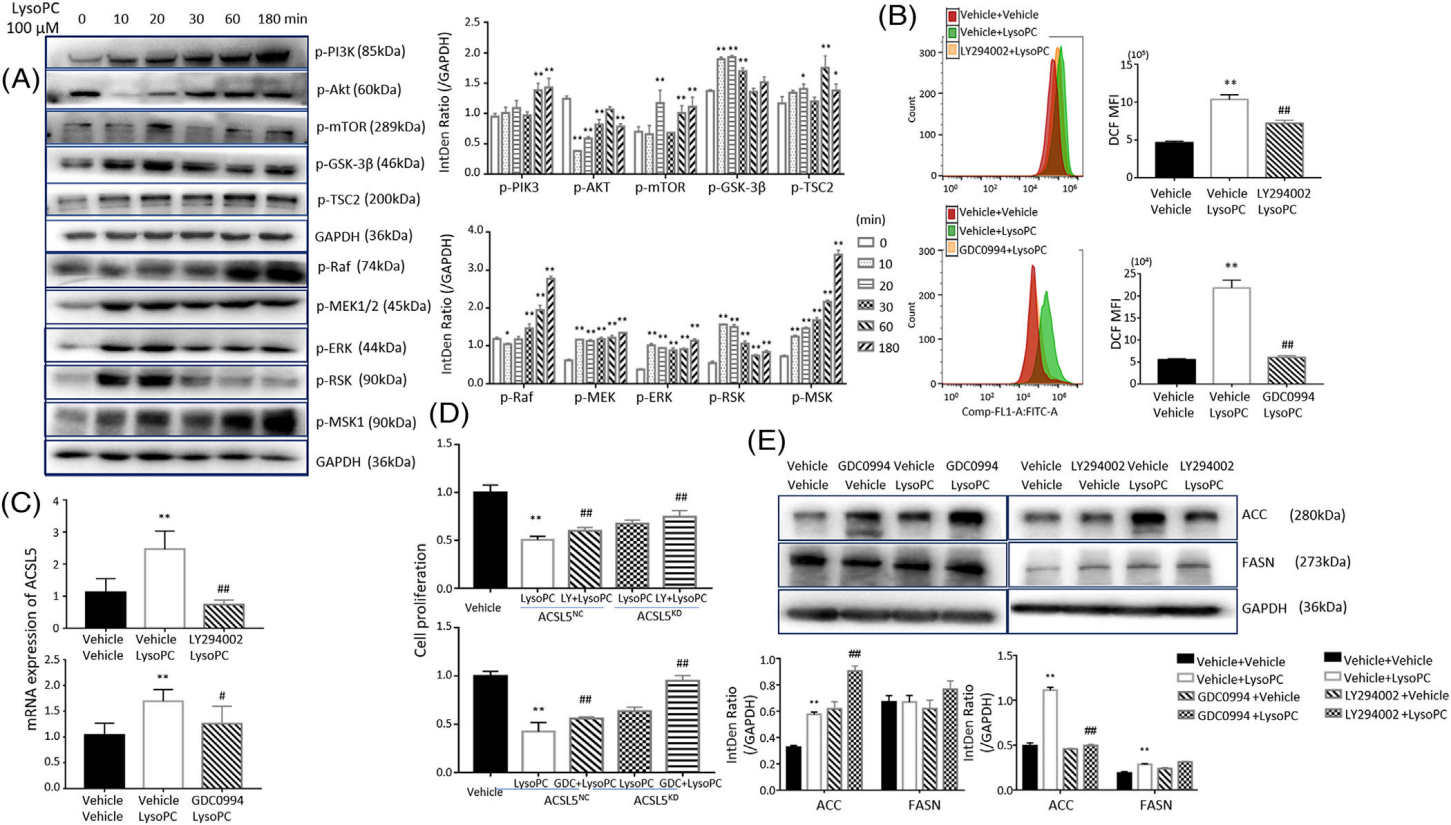
Relationship between phosphorylation of intracellular signal kinases and lysophosphatidylcholine (lysoPC) effects through long‐chain acyl‐coenzyme A synthases 5 (ACSL5). Levels of phosphorylated (p‐) phosphoinositide 3‐kinase (PI3K), Akt, mTOR, GSK‐3β, TSC2, Raf, MEK, ERK, RSK and MSK proteins were measured in SPC‐A1 cells 6 h after treatment with lysoPC. Regulatory effects of PI3K and ERK in reactive oxygen species (ROS) production quantified by DCF fluorescence intensity (B), ACSL5 gene expression (C) and cell proliferation rate (D) were assayed in SPC‐A1 cells pretreated with PI3K inhibitor LY294002 or ERK inhibitor GDC0994 for 2 h and followed by lysoPC treatment for 6 h (*n* = 6) or in ACSL5*^NC^* or ACSL5*^KD^* cells pretreated with inhibitors of PI3K and ERK for 2 h (*n* = 6). Protein levels of ACC and FASN in SPC‐A1 cells pretreated with vehicle or PI3K/ERK inhibitors were measured 6 h after lysoPC treatment (E). * and ** or # and ## stand for the *p*‐values less than .05 and .01, respectively, as compared with cells treated with vehicle or lysoPC

Of transcriptomic expression profiles and RNA‐seq pathway enrichment (Figure S6H), we noticed alternations of signal pathway genes induced by exogenous lysoPC and investigated dynamic changes of some signal pathway genes during 180 min after being treated with lysoPC at 100 μM. We found that phosphorylated protein levels of PI3K significantly elevated at 60–180 min, mTOR and TSC2 at 20–180 min, GSK3b at 10–30 min, MEK, ERK and RSK from 10 min and on, as well as Raf and MSK in a time‐dependent manner (Figure 6A, *p* < .05). We detected changes in PI3K and MAPK isoform mRNA (e.g. PI3KCA, PI3KC2A, PI3KCB, PI3KC2B, PI3KC3, PI3KCG, PI3KC2G, PI3KCD, PI3KR1, PI3KR2, PI3KR3; MAPK1, MAPK3, MAPK4, MAPK6, MAPK7, MAPK8, MAPK9, MAPK11, MAPK12, MAPK13, MAPK14, MAPK15, MAP2K1, MAP2K2 and MAP3K1) after cells were stimulated at different concentrations of lysoPC and found PI3KCA, PI3KC2B, PI3KCG, PI3KCD, PI3KR1, MAPK6, MAPK8, MAP2K1 were up‐regulated, while PI3KC3, PI3KC2G, PI3KR2, PI3KR3, MAPK1, MAPK7, MAPK15, MAP2K2 and MAP3K1 were down‐regulated (Figure S6I,J). There was no significant change in PI3K, AKT and MAPK isoforms observed after ACSL5*^KD^* (Figure S7A–C). The protein expression levels of PI3K, Akt, mTOR and ERK were also examined (Figure S7D).

Inhibitors of PI3Kα/δ/β (LY294002) at 50 nM for 2 h and ERK1/2 (GDC0994) at 10 nM for 2 h could prevent lysoPC‐induced over‐production of ROS (Figure 6B). LY294002 also inhibited lysoPC up‐regulated expression of ACSL5 mRNA in ACSL5*^NC^* cells (Figure 6C) and increased lysoPC‐reduced cell proliferation in ACSL5*^KD^* cells (Figure 6D). The ERK inhibition significantly increased the expression of ACC protein, rather than the PI3K inhibition (Figure 6E, *p* < .01). Inhibiting ERK and PI3K pathways failed to influence the expression of FASN. Inhibition of mTOR with rapamycin (500 nM, 24 h) increased lysoPC‐induced SPC‐A1cell death (Figure S7E) and ROS production (Figure S7F) and reduced the expression of ACSL5 mRNA (Figure S7G). We speculated that GSK‐3β and TSC2 were activated and ultimately regulated SPC‐A1 cell proliferation through mTOR (Figure S7H), similar to the interaction of GSK3, TSC2 and mTOR in other studies.^28^

**FIGURE 7:**
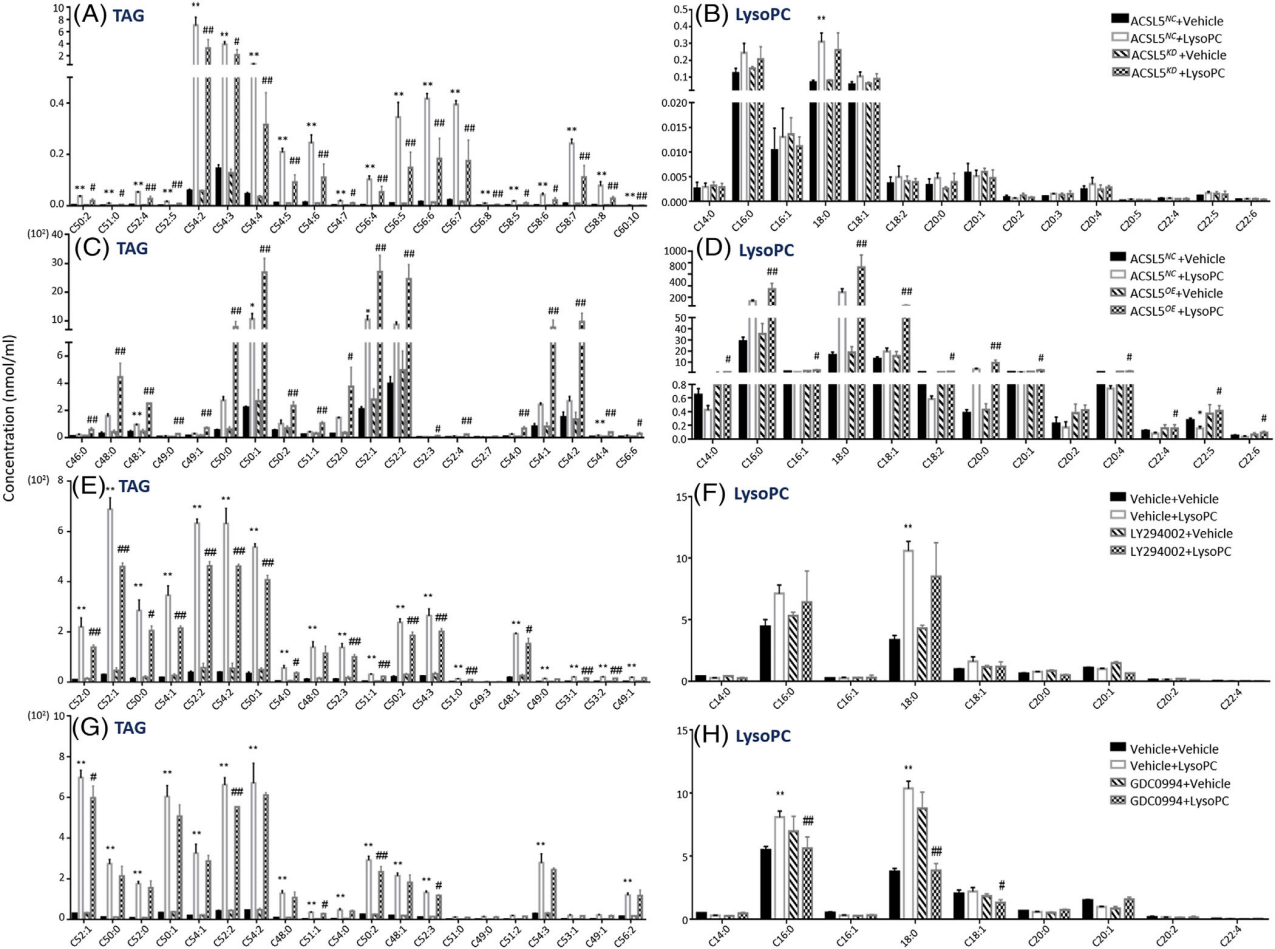
Roles of long‐chain acyl‐coenzyme A synthases 5 (ACSL5) and phosphoinositide 3‐kinase (PI3K) and ERK signal pathways in lipidomic alterations induced by lysophosphatidylcholine (lysoPC). Regulatory effects of ACSL5 and PI3/K/ERK signal pathways in lysoPC‐influenced triacylglycerol (TAG)‐lysoPC balance were investigated, by measuring TAG (A) and lysoPC (B) in ACSL5*^NC^* or ACSL5*^KD^* cells treated with vehicle or lysoPC, TAG (C) and lysoPC (D) in ACSL5*^NC^* or ACSL5*^OE^* cells treated with vehicle or lysoPC, TAG (E) and lysoPC (F) in SPC‐A1 cells pretreated with vehicle or LY294002 and TAG (G) and lysoPC (H) in SPC‐A1 cells pretreated with vehicle or GDC0994 (*n* = 6 per group). * and ** or # and ## stand for the *p*‐values less than .05 and .01, respectively, as compared with cells treated with vehicle or lysoPC

To uncover the roles of ACSL5 and PI3K and ERK signal pathways in lipidomic alterations induced by lysoPC, we measured lipidomic profiles in ACSL5*^NC^* or ACSL5*^KD^* cells or SPC‐A1 cells pretreated with PI3K or ERK inhibitors for 24 h. The intracellular levels of TAG elements in ACSL5*^KD^* cells were lower than that from ACSL5*^NC^* cells after lysoPC stimulation (Figure 7A). Levels of lysoPCs 18:0 elevated in ACSL5*^NC^* cells, rather than in ACSL5*^KD^* (Figure 7B). In ACSL5*^OE^* cells, exogenous lysoPC significantly increased levels of TAG (Figure 7C, *p* < .05) and lysoPC (Figure 7D, *p* < .05). We noticed that SPC‐A1 cells pretreated with LY294002 had less accounts of TAG elements than cells treated with vehicle after being challenged with lysoPC (*p* < .05 or less, Figure 7E), although still higher than cells without lysoPC, while had little effects on lysoPCs (Figure 7F). Pretreatment with GDC0994 partially prevented lysoPC‐increased intracellular levels of some TAG elements (Figure 7G) and lysoPC elements (Figure 7H). Our data indicate that PI3K and ERK signal pathways partially contribute to the maintenance of intracellular contents of TAG and lysoPCs. The results that exogenous lysoPC increased intracellular levels of TAGs and lysoPCs indicate that the conversion of diacylglycerol and fatty acyl‐CoA to triacylglycerol may be activated. Diacylglycerol O‐acyltransferase 1 (DGAT1) as a limiting enzyme of TAGs plays important roles in conversion to TAGs and transfer from acyl CoA to retinol. We found that the inhibition of DGAT1 by PF‐04620110 at 5 μM for 24 h could worsen lysoPC‐induced low cell proliferation rather than DGAT2 (Figure S7I and S7J) and hyper‐production of intracellular ROS (Figure S7K,L).

## 3 DISCUSSION

Clinical lipidomics is an important tool to identify disease‐specific biomarkers and therapeutic targets.^21^, ^22^ Our previous studies demonstrated that circulating levels of lipid elements varied among lung cancer subtypes and clinical phenome severities.^19^ By integrating lipidomic with phenomic profiles, lipid elements and corresponding metabolite panels were found to have the potential of lung cancer subtype biomarkers, for example, PE (36:2 and 18:0‐2) as SCLC specific and lysoPC (20:1 and 22:0) and PC (19:0 and 21:2) as ADC specific. The length, glycerol substitution site and saturation status and attached position of FA vary among disease stages and severities. Serum levels of lysoPC (C26:0 and C26:1) and PC (C42:4 and C34:4) altered obviously in newly diagnosed, stage I non‐SCLC patients.^29^ Disorders of lipid metabolism‐associated gene expression existed in various single cell types measured by single‐cell RNA sequencing of different lung cancer subtypes at the early‐stage, accompanied by alternations of glycerophospholipid metabolism.^30^ The lipid element panel (lysoPC 16:0, 18:0 and 20:4; PC 16:0/18:1, 16:0/18:2, 18:0/18:1, 18:0/18:2 and 16:0/22:6; and triglycerides 16:0/18:1/18:1) was selected as the screening biomarkers in a prospective clinical cohort study and found to have the disease specificity for early‐stage lung cancer.^30^ Most clinical studies on measurements of circulating lipids were retrospective and had comparisons of lipid elements and lipidomic profiles between lung cancer patients and healthy. The present study designed multi‐dimensional and layer prospective clinical studies on values of altered lysoPC in lung cancer, by multi‐comparisons between lung cancer patients and healthy, among lung cancer subtypes, between lung cancer and lung inflammation and between circulation and tissue. Our data evidenced that low levels of lysoPC 16:0 and 18:0‐2 in lung cancer represented the specificity, as compared with healthy and non‐cancer patients. Chronic lung inflammation and exacerbation play important roles in lung carcinogenesis and transit of lung epithelia into cancer and co‐exist with cancer cells during development.^31^, ^32^ Compared with lung cancer, levels of lysoPC16:0, 16:1,18:0, 18:1 and 18:2 species were higher in acute pneumonia and COPD. Studies have shown that lysoPC22:4 and 22:6 had anti‐inflammatory effects while lysoPC16:0,18:0 and 18:1 were pro‐inflammatory.^33^ Low levels of lysoPCs in the circulation and lung tissue indicate that lysoPCs have direct and/or indirect effects on lung cancer proliferation, although the exact molecular mechanisms remain unclear.

Our results demonstrated that lysoPC directly inhibited lung cancer cell proliferation, influenced bio‐behaviours and reprogramed intracellular lipid metabolisms. LysoPC directly changed the morphologic surface of melanoma cells and impaired cellular migratory ability on fibronectin and lung metastasis‐like lesions, by reducing integrin very late antigen‐4 ‐mediated binding to vascular cellular adhesion molecule‐1 and P‐selectin‐dependent interaction with activated platelets.^14^ Our data showed a strong correlation of low lysoPC levels between plasma and lung cancer tissues, which may, at least partially, contribute to the imbalance of soluble *N*‐ethylmaleimide‐sensitive factor attachment protein receptor assembly‐disassembly, loss of membrane fusion and disorder of protein maturation and secretion in cancer cells.^34^ LysoPC has diverse biological and cytotoxic effects, highly dependently upon cell types, mature degrees and exposure time. Exogenous lysoPC could permeabilize the membrane of normal human fetal lung fibroblasts to increase the penetration of small molecules,^35^ probably associated with immature cell development. LysoPC at high doses increased the migration of breast cancer cells with high expression of autotaxin.^36^ We for the first time screened dose‐ and time‐effects of exogenous lysoPC once or continuous exposures on human airway epithelia and various lung cancer cells up to 200 μM for 48 h, which is an abundant extracellular lipid concentration in the body.^37^ Our data evidenced that the sensitivity of lung cancer cells in response to lysoPC varied at different schedules, of which the continuous exposure of lysoPC reduced proliferation, differentiation and movement of SPC‐A1 and increased the rate of cell death clearly. LysoPC pretreatment inhibited lung cancer cell growth in an animal model and changed patterns of lipidomic profiles.

Exogenous lysoPC altered transcriptomic profiles of lung cancer cells, of which ACSL5 was identified and validated as the target gene in lysoPC‐induced lung cancer cell hypoplasia and as a risk factor for lung cancer patient survival. ACSL5 was found to regulate cancer cell sensitivity in response to metabolites.^4^, ^6^ Our data based on transcriptomic profiles of target cells illustrated the altered fatty acid metabolism as the most affected pathway by lysoPC. Of those fatty acids metabolism‐associated and/or specific genes, lysoPC induced obvious over‐expression of ACSL family member genes, for example, ACSL1, 3 and 4 at 6 h maximally, while ACSL5 over‐expressed continuously during 24 h. Of ACSLs, ACSL5 was considered as the target gene, although lysoPCs may stimulate ACSL isoform activations and promote isoform‐specific biological processes, for example, ACSL1‐involved pro‐inflammation, ACSL3‐regulated androgen responsiveness or ACSL4‐and ACSL5‐related tumour suppression.^4^ In conditions of down‐regulated ACSL genes, lung cancer cells lost the sensitivity to lysoPC‐inhibited effects obviously. Patients with high‐expressed‐ACSL5 lung cancers or ADC had high overall survival rates, especially the high progression‐free survivals in ADC and SCC. Of 27 ACS family members for catalyzation of FA into short, medium, long and super‐long‐chain FA,^38^ the ACSL family mainly contributes to the formation of fatty acyl‐CoAs as key regulatory molecules and metabolic intermediates in biological processes, by activating the long‐chain FA.^39^ The ACSL family members have subset‐specific regulatory roles in tissue‐ or cell‐specific expression patterns. Regulatory roles of ACSL subtypes are independent and vary among tissue types and locations, of which products regulate associated metabolic enzymes and signalling pathways for oxidization to provide cellular energy and integration into acylated proteins and complex lipids, such as triacylglycerols, phospholipids and cholesterol esters.^39^, ^40^ ACSL5 mRNA expression up‐regulated in the basal, ciliated club, Goblet and mucous epithelia of ADC patients (vs. healthy), especially in ciliated, club and Goblet cells which differed from patients with COPD, IPF, SSC and para‐cancer tissues. Like other cancers, there are a number of inflammatory cells and mediators in the microenvironment of lung cancer, a progressive process of local fibrosis around cancer cells and a systemic change of an immune function.^32^, ^41^, ^42^, ^43^ Different from lung cancer, ACSL1‐5 is highly expressed in ATI of COPD as the representative of lung chronic inflammation, ACSL1 and 3 in ATI and basal cells of IPF as fibrosis and ACSL4 in ATI cells of SSC as a systemic immune disorder.

ACSL5 plays an important role in lysoPC‐induced metabolism regulatory networks, mitochondrial dysfunction and fatty acid reprogramming. ACSL5 is localized mainly in the mitochondria and regulates fatty acid oxidation and mitochondrial respirometry, which varied among ACSL5 gene polymorphisms and protein isoforms and influenced the body's metabolism.^44^, ^45^ In addition to ACSL5‐regulated conversions from long‐chain FFAs to acyl‐coenzyme A, FA intake and TAG synthesis, ACSL5 participates in a complex process of cell bio‐functions, for example, intestinal stem cell renewal, pancreatic cancer cell protein‐protein interaction network, enterocyte mitochondrial mortalin expression and cell apoptosis.^8^, ^9^, ^45^, ^46^, ^47^ Our present study furthermore demonstrated that ACSL5 plays important role in the control of lung cancer cell sensitivity to exogenous lysoPC by reshaping participants of transcriptional regulators. Of ACSL5‐associated lysoPC‐sensitive genes, the majority contribute to FA metabolism and mitochondria function. ACSL5 acted as a switch between FA degradation and biosynthesis, by which lysoPC altered FA elongation, unsaturated FA biosynthesis, glycerolipid and glycerophospholipid metabolism, inositol phosphate metabolism and phosphatidylinositol signalling system by ACSL5‐regulated FA degradation. Those lipid metabolism pathways were mainly changed in human lung cancer tissues and proposed to support cell proliferation and malignancy.^48^ It seems that exogenous lysoPC may switch on ACSL5 regulation, remodelling synthesis, elongation and desaturation of FAs and changing cell membrane biosynthesis and proliferation since lung ADC cells are sensitive to FA desaturation.^49^ ACSL5‐downregulated lung cancer cells had less or no sensitivities in response to lysoPC, including exogenous lysoPC‐induced alterations of mitochondrial morphology and function, long‐chain FA mitochondrial beta‐oxidation, ROS productions and those lipid metabolisms. It indicates that ACSL5 plays a role in lysoPC‐remodeled lipid metabolisms, evidenced by the fact that levels of lipids are reduced in ACSL5‐downregulated cells and increased in ACSL5‐overexpressed cells. Previous studies stated that cell death caused by ROS was closely related to ferroptosis and that ACSL4 and lysophosphatidylcholine acyltransferase 3 were also important factors in the process of ferroptosis.^50^ The extent of intracellular lipid peroxidation and associated ferroptosis is dependent upon the abundance and localization of polyunsaturated FA.^51^ ACSL1, 3 and 5 were reported to be associated with ferroptosis‐associated disease,^52^ and ACSL5 was also associated with apoptosis.^53^

Multiple intracellular signal pathways are involved in lysoPC‐altered lipid metabolism and proliferation of lung cancer cells. LysoPC mediates a variety of biological functions, including induction of chemotaxis, production of inflammatory factors, oxidative stress and apoptosis, by activating multiple downstream signalling pathways, such as PI3K, MAPK, NF‐κB, G protein‐coupled receptors and Toll‐like receptors.^54^, ^55^ Biological functions of lysoPC vary among cell types, promoting atherosclerotic plaque formation and inflammatory reaction, enhancing anti‐infection response, regulating blood glucose and affecting tumour invasion and metastasis.^55^ LysoPC inhibited endothelial cell migration and healing of arterial injuries partially through the activation of PI3K p110α and p110δ isoforms and promoted phagosome maturation and production of inflammatory mediators through PI3K and p38MAP.^56^, ^57^ LysoPC activated the ERK pathway to regulate the production of ROS and Ca^++^ in mitochondria.^58^ In lung cancer cells, lysoPC could elevate mRNA and protein expression of PI3K‐ and ERK‐signal pathways associated with 10 kinases in lysoPC‐treated cells, presenting multi‐kinase‐based molecular mechanisms (Figure S6I,J). In addition to the interaction among those signal pathways, isoforms of each have their own biological functions, to functionally coordinate and compensate for one another and control cell bio‐behaviours, proliferation and responses to challenges or drugs.^59^, ^60^ We noticed that lysoPC‐stimulated expressions of 11 PI3K isoforms and 15 MARK isoforms varied, of which PI3KC2G, PI3KR2, PI3KR3 or MAPK15 declined in a dose‐dependent pattern. LysoPC‐altered those signal pathways regulated ACSL5 expression, since down‐regulation of ACSL5 failed to influence mRNA expression of those kinase isoforms (Figure S6I,J), while the inhibition of PI3K and ERK prevented lysoPC‐increased ACSL5 expression. Long‐term energy stress increased saturated FA loss and unsaturated FA gain, fatty acid oxidation, ACSL gene hypo‐expression and AMPK and PI3K hyper‐expression in yaks.^61^ The PI3K inhibition prevented ACSL gene hyper‐expression, fat accumulation, oxidative stress and insulin resistance through the reduction of insulin signalling in mouse hepatocytes.^62^ We found that PI3K or ERK inhibition could decrease lysoPC‐induced lung cancer cell death by reducing ROS production and increasing acetyl‐CoA carboxylase activation.

ACSL5 plays a critical role in lysoPC‐induced lipid metabolism in lung cancer cells, of which TAGs‐lysoPC bi‐directional metabolites were the central balance. TAGs and lysoPC are important metabolites transferred from DAGs and changed with various diseases, locations and challenges. The circulating levels of TAGs increased, while lysoPCs decreased with human age, which can be altered during lifespan regulation and the development of age‐related diseases.^62^ Data from clinical trials illustrated that the intervention with medium‐chain triglycerides increased blood levels of lysoPCs (e.g. 16:0, P‐18:0, P‐18:1[9Z], 20:2[11Z,14Z] and 22:5[4Z,7Z,10Z,13Z,16Z]), as the part of positive effects on cognitive ability in mild to moderate Alzheimer's disease patients without APOE4.^63^ The present study demonstrated that exogenous lysoPCs increased intracellular contents of TAGs, probably by incorporating into cellular phospholipids, converting from FAs origin PA and DAGs, decreasing cholesteryl ester synthesis and secretion and/or promoting TAG synthesis and secretion (Figure S4A). Our data from ACSL5 knockdown and overexpression evidenced that ACSL5 was crucial for the control and maintenance of the sensitivity of lung cancer cells to exogenous lysoPC by reprogramming and regulation of TAG‐lysoPC metabolism. Down‐regulation of ACSL5 or PI3K/ERK inhibition resulted in the loss of metabolism sensitivity to lysoPC in lung cancer cells, through remodelling patterns and efficiency of lipid metabolism, influencing mitochondrial function and respiration or leading to TAG accumulations by increasing TAG synthesis or delaying TAG absorption, similar to the findings based on the ablation of ACSL5 in the in vivo model and on the regulation of ACSL5 in the TAG synthesis.^64^, ^65^

Limitations of the present study: although a large number of samples were collected to verify the changes in lysoPC in humans, there was then no careful delineation of the prognosis, treatment effect or choice of the treatment regimen for the source of the samples and whether the level of lysoPC correlates with the expression of ACSL5 in tumour tissues. Further research is required to determine the location of lysoPC in lung tissues, particularly in malignant or paraneoplastic tissues. In technology, GAPDH is a key gene in glycolysis and its expression in metabolism will be affected by many factors, so it needs to be carefully considered as an internal reference. Additionally, future research needs to make improvements in the limited number of animal experiments used to confirm the effect of the target gene ACSL5 in vivo.

**FIGURE 8:**
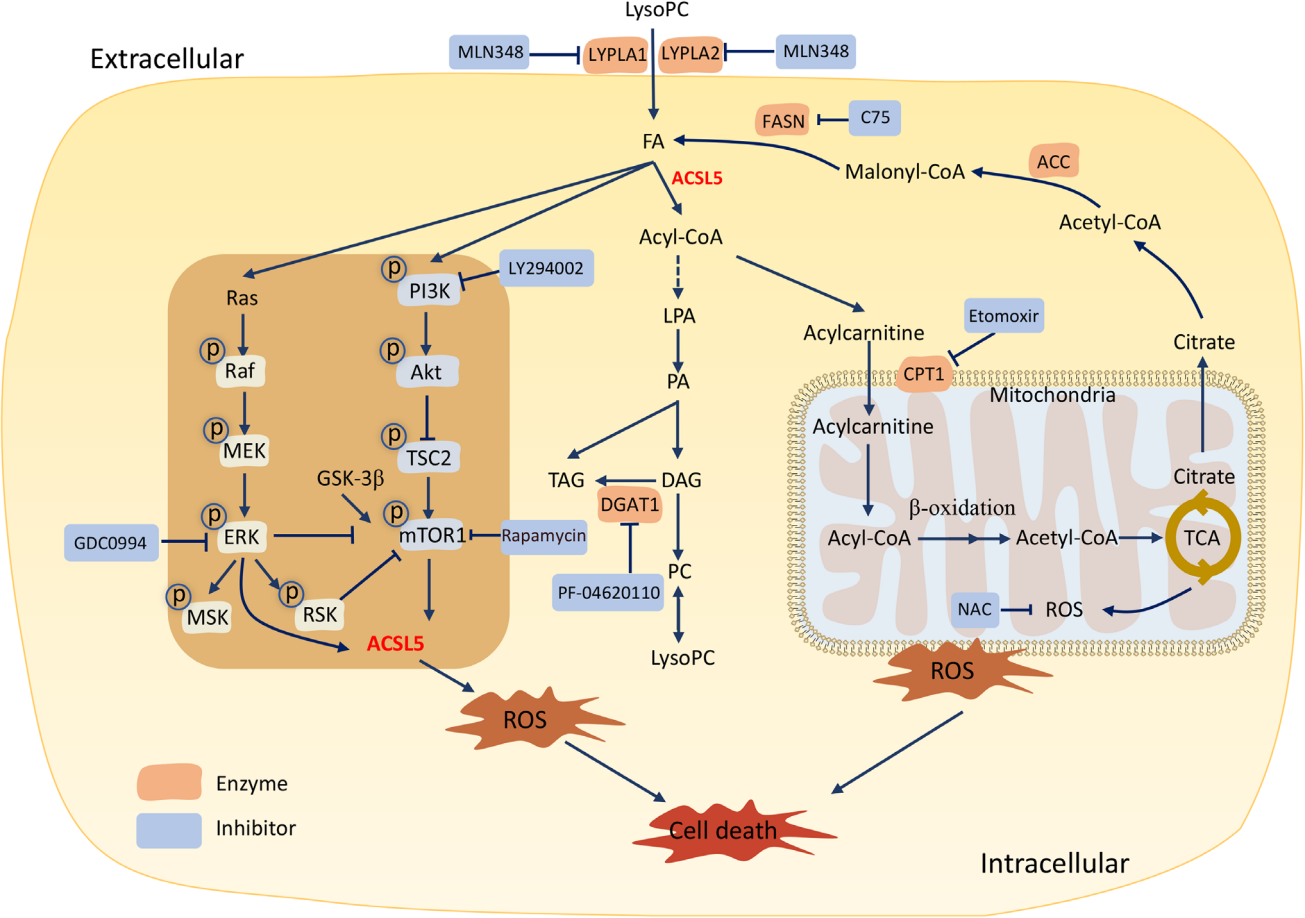
Molecular mechanisms of exogenous lysophosphatidylcholines (lysoPCs)‐inhibited lung cancer cell proliferation. Exogenous lysoPC reprograms lipid metabolisms by increasing the accumulation of fatty acids (FA) and activating acyl‐CoA‐dominated metabolic mode under the control and regulation of long‐chain acyl‐coenzyme A synthases 5 (ACSL5). LysoPC up‐regulates the expression of ACSL5 and activates the ACSL5‐oriented lipid metabolism by promoting the phosphorylation of phosphoinositide 3‐kinase (PI3K)/mTOR and Ras/ERK signal pathways. ACSL5 plays a decisive role in the process during which lysoPC alters mitochondrial morphology and function as well as reactive oxygen species (ROS) over‐production. Exogenous lysoPC inhibited lung cancer cell proliferation by promoting ACSL5, leading to the disorder of FA degradation and metabolism, mitochondrial dysfunctions and reprogramming of ACSL5/PI3K/ERK‐regulated triacylglycerol (TAG) metabolism. Multi‐dimensions, layers and interactions of intracellular factors and signal pathways consist of a complex regulation of lung cancer cell sensitivity to lysoPC, evidenced by inhibition of involved signal pathways

Collectively, this study evidences that the low level of lysoPCs can be one of the characteristics in patients with lung cancers with high specificity and exogenous lysoPCs inhibit lung cancer cell proliferation and growth, accompanied by the reprogramming of lipid metabolisms (Figure 8). Of FA metabolism and ACSL family members, ACSL5 over‐expressed in lung epithelia with structural, proliferative, cleaning and secretory functions in patients with lung cancers. Exogenous lysoPC inhibited lung cancer cell proliferation by promoting ACSL5, leading to the disorder of FA degradation and metabolism, compromise of mitochondrial morphology and functions and reprogramming of ACSL5/PI3K/ERK‐regulated TAG metabolism. Multi‐dimensions, layers and interactions of intracellular factors and signal pathways consist of a complex regulation of lung cancer cell sensitivity to lysoPC, evidenced by inhibition of involved signal pathways (Figure 8). Thus, our data provide new insights for the discovery and development of reprogramming metabolisms and metabolites as a new strategy of lung cancer precision medicine.

## 4 METHODS

### 4.1 Clinical studies

The healthy subject was characterized by the population of health screening without lung diseases, respiratory disease symptoms and signs and metabolic diseases such as hypertension, hyperlipidemia or diabetes. Patients with lung cancer were recruited according to the Union for International Cancer Control lung cancer definition and histopathological diagnostic criteria,^66^ and COPD was recruited according to the Global Initiative for Chronic Obstructive Lung Disease diagnosis.^67^ Acute pneumonia is diagnosed with new localized chest signs and new pulmonary infiltrates on chest radiograph due to acute symptoms such as dyspnea, cough and fever without other obvious causes.^68^, ^69^ Blood was harvested from recruited patients and healthy control individuals in the early morning, to avoid the influence of diets and drugs. Clinical studies included circulating plasma profiles of lipidomics in discovery study, validation study, subtype validation and lung disease validation, as well as lung tissue levels of target lipid panels, as shown in Figure 1A. The first case‐control study was designed to define whether lipidomic profiles in the circulation changed between healthy and patients with lung cancer. Plasma samples (*n* = 33) were collected from 8 healthy subjects and 25 lung cancer patients, including ADC (*n* = 8), SCC (*n* = 9) and SCLC (*n* = 8). The second case‐control study was designed to validate the findings from the first case‐control study in a large population and the difference among subtypes of lung cancer, plasma samples were harvested from a healthy individual (*n* = 290) and ADC (*n* = 63), SCC (*n* = 21) and SCLC (*n* = 12). The third case‐control study was designed to estimate the disease specificity of the lysoPC panel, plasma levels of lysoPCs in lung cancer (*n* = 96) were compared with those in acute pneumonia (*n* = 120) and COPD (*n* = 66), referred with healthy subjects (*n* = 290). The fourth case‐control study was designed to validate levels of lysoPC in lung cancer and para‐cancer tissues (*n* = 10 pairs) from the operation. Each of the studies has ethical permission proved by the Ethical Committee of Fudan University Zhongshan Hospital for clinical discovery and validation studies (B2018‐187, B2019‐197[2], respectively). The study complied with the principles of the Declaration of Helsinki. Clinical data of patients according to previous scoring criteria^6^ were presented in Table S5.

### 4.2 Cell proliferation

In order to screen lysoPC‐sensitive lung epithelial cells, the cell proliferation of large cell human lung carcinoma cells (NCI‐H460 and NCI‐H661) that expresses easily p53 mRNA detection, non‐SCLC cells (NCI‐H1299), lung ADC (A549 and NCI‐H1650), SCLC cells (NCI‐H1688), gene‐edited lung ADC epithelia (A549*^p53−^*), lung bronchial ADC epithelia (SPC‐A1 cells) and normal bronchial epithelia (HBE135‐E6E7) were measured using CCK8 (C0037, Beyotime, Shanghai, China) at 3, 6, 12, 24 and 48 h after treatment with exogenous lysoPC (L1381, Sigma‐Aldrich, MO, USA) at concentrations of 25, 50, 100 or 200 μM. Cells were cultured in full media consisting of RMPI 1640 (KGM31800‐500, KeyGEN, Jiangsu, China) containing 10% fetal bovine serum (F8318, Sigma‐Aldrich, MO, USA), penicillin 100 U/ml and streptomycin 100 mg/ml and in the cell incubator at 37°C with 5% CO_2_.

### 4.3 Quantitative real‐time polymerase chain reaction

TRIzol reagent (15596026, Invitrogen, USA) was used for RNA extracted according to the manufacturer's protocol and cDNA was synthesized with PrimeScript RT Master Mix (RR036A; Takara, Japan). Real‐time polymerase chain reaction (PCR) was performed by TB Green Premix Ex Taq (RR420A; Takara) with ABI 7000 PCR instrument (Eppendorf, Hamburg, Germany) by two‐step program parameters. The expression level of the target gene is normalized by the ACTB housekeeping gene and the expression level is calculated by the comparative method (2^^−ΔΔCt^).

### 4.4 Western blots

Cell protein was obtained using RIPA buffer (P0013C; Beyotime) supplemented with protease inhibitor and phosphatase inhibitor (P1082 and P1087; Beyotime). The protein was separated by 8% sodium dodecyl sulphate‐polyacrylamide gel electrophoresis and transferred to the enhanced chemiluminescence nitrocellulose membrane. The membranes were first blocked and then incubated with various primary antibodies followed by secondary antibodies (horseradish peroxidase‐conjugated antibody). Primary antibodies were used, including p‐PI3K (17366S; CST, MA, USA), p‐Akt (4060; CST), p‐mTOR (2974T; CST), p‐GSK‐3β (5558; CST), p‐TSC2 (23402S; CST), p‐Raf (9427S; CST), p‐MEK(9154S; CST), p‐ERK (4370S; CST), p‐RSK (11989S; CST), p‐MSK(9595S; CST), CPT1A (ab128568; Abcam, Cambridge, UK), CPT1B (ab134135; Abcam), BIP (3177; CST), CHOP (2895; CST), ACC (12589T; CST), FASN (12589T; CST), PI3K (3358; CST), Akt (4685; CST), mTOR (2983; CST), ERK (4695; CST) and GAPDH (AF1186; Beyotime). HRP‐linked anti‐mouse or rabbit IgG (7076 or 7074; CST) was used as secondary antibodies. The immunoreactivity was shown by Immobilon (P90719; Millipore, MA, USA). The gray value of the western bolt calculated by ImageJ.

### 4.5 Liquid chromatography‐tandem mass spectrometry

Lipid was extracted from the cell suspension of trypsin‐digested adherent cells, after centrifugation to obtain the cell pellets were centrifugated, collected and added with 900 μl of water and vertex for 10 s. the cell membrane was permeabilized with ultrasound for 30 s and added with 1 ml chloroform and 2 ml methanol for one minute and with 1 ml chloroform, 1 ml water and 10 μl of internal standard (Internal Standards Kit for Lipidyzer Platform 5040156; AB SCIEX, MA, USA and 55107; Sigma‐Aldrich, MO, USA) for additional one min, respectively. The solution was centrifuged for 15 min at 2500 rpm. The lipid‐containing organic layer at the bottom with nitrogen was dried and finally reconstituted with 200 μl chloroform/methanol 1:2 containing 10 mM ammonium acetate.

Plasma lipids were extracted as reported previously.^70^ In brief, 20 μl of plasma was taken into a 1.5 ml Eppendorf tube, followed by adding 350 μl of pre‐cooled isopropanol and 10 μl of internal standard. After mixing, samples were placed for 10 min at room temperature (RT) and overnight at ‐20°C. Samples were centrifugated at 1200 rpm for 20 min, of which 200 μl of supernatant was collected and centrifuged for 20 min. After then, 10 μl from each sample was taken and mixed as a quality control.

Using liquid chromatography‐tandem mass spectrometry (QTRAP 5500; AB SCIEX), the ion source mode was electrospray ionization and the mass analyzer was a Q‐Trap running in multi‐reaction monitoring working mode. The analytical column was performed on the Acquity UPLC BEH HILIC column (100 × 2.1 mm, 1.7 μm). The mobile phase A was 95% acetonitrile solution and B was 50% acetonitrile solution, both containing 10 mmol/L ammonium acetate. The injection volume of both positive and negative ion modes was 4 μl. It should be noted that the blood samples from the first case‐control study were tested according to previous methods.^6^ The level of lysoPC varies due to different detection methods.

### 4.6 Cell bio‐behaviours

Cell biological behaviours, for example, proliferation, death, movement and differentiation, were measured by the live cell imaging technology Cell‐IQ (Chip‐Man Technologies, Tampere, Finland), to obtain the dynamic change process of the cells, taking pictures every 2 h. This technology was operated through the Manual Tracking plug‐in produced by Fabrice Cordelieres (Institut Curie, Orsay, France). This vision technique can quantitatively analyze cell function and morphological parameters.

### 4.7 Target RNA interference

The valid sequences of small interference RNA (siRNA) are as below: ACSL5: sense‐GCUUGUUACACGUACUCUATT, antisense‐UAGAGUACGUGUAACAAGCTT; negative control: sense‐UUCUCCGAACGUGUCACGUTT, antisense‐ACGUGACACGUUCGGAGAATT; ACSL4: sense‐ CCAAGUAGACCAACGCCUUTT, antisense‐ AAGGCGUUGGUCUACUUGGTT were screened and selected. Lipofectamine 2000 (11668019; ThermoFisher Scientific, MA, USA) was added to siRNA or negative control and then placed for 20 min at RT. After 8 h incubation at 37°C incubator, SPC‐A1 cells were transfected with siRNA ACSL5 for experiments. Transfection of ACSL5 overexpression plasmid (101000046; Polyplus, NY, USA) was performed by jetPRIME.

### 4.8 Seahorse real‐time cell metabolic analysis

The Seahorse XF (Agilent) was used to detect cell mitochondrial stress and obtain OCR and ECAR data. SPC‐A1 cells were infected with siRNA ACSL5 for 24 h, treated with vehicle or lysoPC for 6 h and placed in a plate containing 1640 medium and 10% serum. After overnight incubation, oligomycin 1 μM, carbonyl cyanide 4‐trifluoromethoxy‐phenylhydrazone 2 μM and rotenone 50 μM were added to each well.

### 4.9 Cell bulk RNA sequencing

RNA integrity was assayed using RNA Nano 6000 Assay Kit by Bioanalyzer 2100 System (Agilent Technologies, CA, USA). mRNA of total RNA was purified using magnetic beads with poly‐T oligonucleotide attached. The purified double‐stranded cDNA was screened for cDNA of about 370–420 bp, PCR amplification was performed, AMPure XP beads (Beckman Coulter, Beverly, USA) were used to purify the PCR product again and the library was obtained. The library was pooled and sequenced using Illumina NovaSeq 6000 platform. To evaluate the prognostic value of the ACSL5 gene in lung cancer patients, Kaplan‐Meier‐plotter (https://kmplot.com/analysis/) was used to analyze the overall survival rate and progression‐free survival rate of lung cancer patients in the GEO, EGA and TCGA database. Based on the results of RNA sequencing, the gene expression values displayed in the current study were the Fragments Per Kilobase per Million mapped reads.

### 4.10 Validation of lung single epithelial ACS family gene expression (scRNA‐seq)

The expression of ACS family members (*n* = 27) in alveolar epithelial types I and II, basal, ciliated, club, Goblet and mucous epithelia was analyzed from datasets (GSE128169, GSE131907 and GSE136831). Data were normalized on basis of the expression of hypervariable genes and integration anchors and integrated. The data were preprocessed through standardization, dimensionality reduction and cell clustering. The annotation label of the Seurat mapping references was used to annotate the data set. The expression of ACSL family genes (*n* = 5) was evaluated in different epithelia and lung diseases, including COPD 15 samples, IPF 15 samples, ADC 15 samples, SSC eight samples, healthy (normal lung tissues) 20 samples and para‐cancer tissue 11 samples. Seven types of airway and lung epithelial cells were characterized and labelled with corresponding cell‐specific genes, as listed in Table S6.

### 4.11 ROS detection

Cells were incubated in a 6‐well plate, stimulated with vehicle or lysoPC at different concentrations for 6 h and digested with trypsin. ROS levels were measured with the kit (S0033S; Shanghai, China), using the fluorescent probe DCFH‐DA. DCFH‐DA itself without fluorescence freely passed through the cell membrane, entered the cell and was hydrolyzed by intracellular esterase to generate DCFH. Intracellular ROS oxidized non‐fluorescent DCFH to generate fluorescent DCF. The intensity of DCF fluorescence was detected as the level of intracellular ROS after DCFH‐DA was diluted with a serum‐free medium at 1:1000. Cells were collected, suspended, incubated for 20 min at 37°C and washed thrice with serum‐free medium, then transferred to flow tubes (BD FACS Aria II, NJ, America) for measurement.

### 4.12 Fluorescence staining and quantification

Cells were fixed with 4% paraformaldehyde for 10 min, then permeabilized for 10 min with 0.5% Triton X‐100 at RT. After three rinses with phosphate‐buffered saline (PBS), ACSL5 (sc‐365478; Santa Cruz Biotechnology, TX, USA) antibodies were incubated overnight and subsequently with Cy3‐conjugated goat anti‐mouse IgG (H+L) (A0521; Beyotime) for 2 h. After counterstaining with DAPI (KGA215; KeyGEN, Jiangsu, China) for 15 min, the cells were observed.

### 4.13 Immunohistochemistry detection

Intracellular ACSL5 proteins were detected and measured by immunohistology. Freshly dissected tissue was fixed in 2% paraformaldehyde for 1 h overnight at RT rinsed, dehydrated in alcohol and cleared twice in xylene. Embed the tissue in a paraffin block. Transfer sections to glass slides. Deparaffinize slides in deparaffinized for three times, 10 min each. Repair tissue sections with PH6.0 citric acid retrieval solution. After cooling, the slices were immersed in 3% H_2_O_2_ for 30 min. After washing, sections were incubated and blocked with 10% goat serum for 30 min at RT. After removing the serum, the primary antibody (PHS9734; Abmart, Shanghai, China) was diluted with 10% goat serum, 50 μl primary antibody was added dropwise to each section and incubated overnight at 4°C, then the secondary antibody (ab205718, Abcam, Cambridge, UK) at RT for 60 min. After hematoxylin nuclear staining, mount the slides with the mounting solution. Observe the antibody staining under a white light microscope.

### 4.14 Measurements of mitochondrial function and morphology

For mitochondrial mass and membrane potential assessment, Mitotracker green and Mitotracker red CMXRos (C1048 and C1049; Beyotime) were used for staining at 37°C for 30 min and assessed by flow cytometry. A High Content Analysis System (Perkin‐Elmer Operetta, MA, USA) was used to extract mitochondrial texture to analyze mitochondrial morphology. Texture characters of mitochondria, that is, signal enhancement ratio (SER) features, were calculated mathematically on basis of Gaussian derivative filters, including eight mode points, edges, ridges, saddles, valleys, light and dark. The mitochondrial morphology was measured by kernel normalization and quantified by calculating the fraction of SER Ridge.^71^ To examine the ultrastructure of cell morphology, cells were imaged under transmission electronic microscopy, using the ImageJ program. The length/width ratios of mitochondria were measured to represent the degree of cellular mitochondrial fragmentation.^72^

### 4.15 Measurement of apoptosis

Trypsinized cell digestion solution to obtain SPC‐A1 cell suspension. After washing with PBS, discard the supernatant and resuspend the cells with Annexin V‐FITC binding solution. Add 5 μl Annexin V‐FITC and 10 μl propidium iodide staining solution (C1062M; Beyotime). Mix gently and incubate at RT for 20 min in the dark. Finally, flow cytometry detection.

### 4.16 SPC‐A1 cell‐derived xenograft mouse models

Female NCr‐nu/nu mice (JSJ, shanghai) of 6–8 weeks of age were used to generate a xenograft model. SPC‐A1 cells (2 × 106) in the logarithmic growth phase were suspended in 100 μl of normal saline and implanted in the flank of the mouse to generate a subcutaneous model. After 10 days, the tumour formation in the armpit was observed. For drug therapy, lysoPC (40 mg/kg/day) was formulated in 100 μl normal saline for intraperitoneal injection. The same volume of normal saline was injected for the control group. For the side effects and toxicity of lysoPC, 10 healthy mice were randomly divided into two groups and injected intraperitoneally with equal amounts of saline and lysoPC (40 mg/kg/day) for 7 days, respectively and mice weight was recorded. On day 8, the liver, brain and lungs of mice were collected for H&E staining and pathological scoring. The scoring criteria were shown in Table S7. For in vivo experiments to validate the role of ACSL5 in lysoPC, we pretreated SPC‐A1 cells with siRNA knockdown of ACSL5 gene or NC and then implanted the treated cells into the armpits of mice, respectively, after 5 days of growth, saline or lysoPC (40 mg/kg/day) was injected intraperitoneally for 7 days.

### 4.17 Statistics

Data after lipid normalization were used and collected for absolute quantification (data with internal standard) and peak area (without internal standard) for relative quantification, using Analyst software. SCIEX OS software was used to quantify and normalized the peak area according to the detection area value of the internal standard (Normalized data = peak area/internal standard detection peak area × internal standard concentration). Ultimately, usable data need to exclude RSD ≤30% (RSD = standard deviation/average value). Cell lipidomic results were standardized according to cellular protein levels. Mice were randomized into groups. Tumour volume = (length × width^2)/2. Tumour size was measured with vernier callipers. Prism 9 statistical software (Graph Pad Software, CA, USA) was used for data analysis, the lipid heat map was analyzed by MetaboAnalyst web (www.metaboanalyst.ca), the lipid gene network map was analyzed by Cytoscape 3.9.0 and IHC mean density was analyzed by Image‐pro plus 6.0 (Mean density = (IOD SUM)/area). Unpaired two‐tailed Student *t*‐tests or one‐way analysis of variance were used to calculate p‐values for bar graphs.

## CONFLICT OF INTEREST

The authors declare that they have no conflict of interest.

## Supporting information

Supporting Information

Supporting Information

Supporting Information

Supporting Information

Supporting Information

Supporting Information

Supporting Information

Supporting Information
