# Supplementary material for: Lysophosphatidylcholine inhibits lung cancer cell proliferation by regulating fatty acid metabolism enzyme long‐chain acyl‐coenzyme A synthase 5
Source: Clin Transl Med. 2023 Jan 13;13(1):e1180. doi: 10.1002/ctm2.1180 (PMC9839868; doi:10.1002/ctm2.1180)
Supplement: Supplementary file 1 — Supporting Information [file CTM2-13-e1180-s002.docx]

Lysophosphatidylcholine inhibits lung cancer cell proliferation by regulating fatty acid metabolism enzyme ACSL5

Linlin Zhang^1*^, Xuanqi Liu^2*^, Yifei Liu^3^, Furong Yan^1,3^, Yiming Zeng^3^, Yuanlin Song^1,2,4^, Hao Fang^5^, Dongli Song^1,2,4^, Xiangdong Wang^1,2,4^

^1^ Department of Pulmonary and Critical Care Medicine, Zhongshan Hospital, Fudan

University Shanghai Medical College, Shanghai;

^2^ Shanghai Institute of Clinical Bioinformatics, Shanghai;

^3^ Center of Molecular Diagnosis and Therapy, The Second Hospital of Fujian Medical University, Quanzhou, Fujian;

^4^ Shanghai Engineering Research for AI Technology for Cardiopulmonary Diseases, Shanghai, China.

^5^Department of Anesthesiology, Zhongshan and Minhang Hospital, Fudan University, Shanghai, China.

*Authors contribute to this article equally as the first author.

Correspondence to:

Xiangdong Wang, MD, PhD, Prof

Email: xdwang@fuccb.com

Hao Fang, MD, PhD

fang.hao@zs-hospital.sh.cn

Dongli Song, MD, PhD, Assoc Prof

Email: songdongli37@126.com

Supplemental Table 1. lipid elements increased or decreased significantly in patients with lung cancer in Discovery study.

|  | ADC VS Healthy | | SCC VS Healthy | | SCLC VS Healthy | |
| --- | --- | --- | --- | --- | --- | --- |
| Lipid name | Fold change | P value | Fold change | P value | Fold change | P value |
| lyaoPC | 0.67 | 0.01 | 0.75 | 0.06 | 0.57 | 0.00 |
| lysoPE | 0.24 | 0.00 | 0.42 | 0.00 | 0.32 | 0.00 |
| lysoPG | 0.81 | 0.47 | 0.61 | 0.11 | 48.88 | 0.25 |
| lysoPI | 0.46 | 0.09 | 0.46 | 0.04 | 1.47 | 0.46 |
| lysoPS | 121.14 | 0.01 | 118.77 | 0.08 | 125.58 | 0.12 |
| PC | 0.86 | 0.24 | 0.85 | 0.23 | 0.93 | 0.47 |
| PE | 1.34 | 0.30 | 1.43 | 0.07 | 1.42 | 0.08 |
| PG | 0.61 | 0.17 | 0.73 | 0.49 | 42.47 | 0.24 |
| PI | 0.86 | 0.36 | 1.01 | 0.97 | 5.24 | 0.17 |
| PS | 85.31 | 0.01 | 26.57 | 0.08 | 43.68 | 0.10 |
| SM | 0.85 | 0.20 | 0.83 | 0.23 | 0.75 | 0.06 |

Supplemental Table 2. lipid elements increased or decreased significantly in patients with lung cancer or non-cancer diseases in disease-specificity validation study.

|  | Lung cancer VS Healthy | | Acute pneumonia VS Healthy | | COPD VS Healthy | |
| --- | --- | --- | --- | --- | --- | --- |
| Lipid name | Fold change | P value | Fold change | P value | Fold change | P value |
| lysoPC(14:0) | 0.72 | 0.00 | 1.05 | 0.38 | 1.43 | 0.00 |
| lysoPC(16:0) | 0.80 | 0.00 | 2.12 | 0.00 | 2.21 | 0.00 |
| lysoPC(16:1) | 0.70 | 0.00 | 1.34 | 0.00 | 1.70 | 0.00 |
| lysoPC(18:0) | 0.78 | 0.00 | 1.77 | 0.00 | 1.73 | 0.00 |
| lysoPC(18:1) | 0.74 | 0.00 | 1.30 | 0.00 | 1.63 | 0.00 |
| lysoPC(18:2) | 0.74 | 0.00 | 1.00 | 0.98 | 1.58 | 0.00 |
| lysoPC(18:3) | 0.59 | 0.00 | 0.74 | 0.00 | 1.11 | 0.23 |
| lysoPC(20:0) | 0.90 | 0.01 | 0.81 | 0.00 | 0.84 | 0.01 |
| lysoPC(20:1) | 0.86 | 0.02 | 1.03 | 0.64 | 0.99 | 0.94 |
| lysoPC(20:2) | 0.72 | 0.00 | 0.92 | 0.08 | 1.05 | 0.37 |
| lysoPC(20:3) | 0.78 | 0.00 | 0.76 | 0.00 | 0.92 | 0.23 |
| lysoPC(20:4) | 0.69 | 0.00 | 0.88 | 0.01 | 0.95 | 0.40 |
| lysoPC(20:5) | 0.77 | 0.00 | 0.51 | 0.00 | 0.49 | 0.00 |
| lysoPC(22:4) | 0.81 | 0.00 | 0.27 | 0.00 | 0.12 | 0.00 |
| lysoPC(22:5) | 0.90 | 0.05 | 0.01 | 0.00 | 0.13 | 0.00 |
| lysoPC(22:6) | 0.81 | 0.00 | 0.35 | 0.00 | 0.19 | 0.00 |

Supplemental Table 3. Up- or down-regulated expressions of transcriptomic DEGs after interfering with ACSL5 and lysoPC treatment

| Gene name | ACSL5*^NC^* + Vehicle Mean | ACSL5*^NC^* + lysoPC Mean | ACSL5*^KD^* + lysoPC Mean | ACSL5*^NC^* + lysoPC/ACSL5*^NC^* + Vehicle Fold change | P value | ACSL5*^NC^* + lysoPC/ACSL5*^KD^*+ lysoPC Fold change | P value |
| --- | --- | --- | --- | --- | --- | --- | --- |
| HES5 | 0.22 | 0.38 | 0.05 | 1.70 | 0.22 | 7.64 | 0.00 |
| PFN1P4 | 1.04 | 1.18 | 0.16 | 1.14 | 0.33 | 7.26 | 0.00 |
| KRT9 | 0.23 | 0.26 | 0.05 | 1.14 | 0.84 | 5.34 | 0.05 |
| SRP14P2 | 0.98 | 1.92 | 0.38 | 1.96 | 0.14 | 5.11 | 0.01 |
| SHC2 | 0.10 | 0.10 | 0.02 | 1.08 | 0.89 | 4.90 | 0.01 |
| PIK3R1 | 0.01 | 0.02 | 0.00 | 1.25 | 0.62 | 4.48 | 0.02 |
| FBXW12 | 0.12 | 0.20 | 0.04 | 1.61 | 0.04 | 4.42 | 0.00 |
| LGR6 | 0.07 | 0.08 | 0.02 | 1.11 | 0.68 | 4.22 | 0.01 |
| NPC1L1 | 0.08 | 0.10 | 0.03 | 1.27 | 0.31 | 3.85 | 0.02 |
| EVI2B | 0.15 | 0.37 | 0.10 | 2.52 | 0.06 | 3.83 | 0.02 |
| PVALEF | 0.07 | 0.27 | 0.07 | 3.75 | 0.04 | 3.63 | 0.02 |
| CPT1B | 0.06 | 0.04 | 0.01 | 0.67 | 0.28 | 3.44 | 0.04 |
| TTC9B | 0.09 | 0.28 | 0.08 | 3.03 | 0.05 | 3.35 | 0.02 |
| CHRNA4 | 0.06 | 0.03 | 0.01 | 0.59 | 0.24 | 3.32 | 0.00 |
| APOL3 | 0.08 | 0.10 | 0.03 | 1.26 | 0.27 | 3.27 | 0.01 |
| OTOF | 0.06 | 0.09 | 0.03 | 1.42 | 0.50 | 3.24 | 0.00 |
| SLC15A3 | 0.03 | 0.10 | 0.03 | 3.33 | 0.02 | 3.11 | 0.03 |
| ARSI | 0.07 | 0.17 | 0.05 | 2.54 | 0.09 | 3.10 | 0.01 |
| C11orf1 | 2.85 | 2.12 | 0.68 | 0.74 | 0.50 | 3.10 | 0.00 |
| SLCO2B1 | 0.15 | 0.13 | 0.04 | 0.85 | 0.72 | 3.08 | 0.01 |
| PRDM12 | 0.33 | 0.38 | 0.12 | 1.14 | 0.64 | 3.04 | 0.03 |
| GNGT2 | 0.60 | 0.47 | 0.16 | 0.79 | 0.63 | 3.02 | 0.00 |
| ELFN1 | 0.23 | 0.43 | 0.14 | 1.84 | 0.01 | 3.00 | 0.00 |
| ASIP | 0.54 | 0.50 | 0.17 | 0.92 | 0.74 | 2.92 | 0.00 |
| COL13A1 | 0.08 | 0.18 | 0.06 | 2.29 | 0.08 | 2.82 | 0.01 |
| TTLL13P | 0.02 | 0.01 | 0.06 | 0.47 | 0.50 | 0.19 | 0.03 |
| WBP1LP2 | 0.31 | 0.44 | 1.01 | 1.40 | 0.43 | 0.19 | 0.05 |
| RNF113B | 0.16 | 0.09 | 0.40 | 0.61 | 0.27 | 0.20 | 0.00 |
| LINC00570 | 0.25 | 0.05 | 0.15 | 0.22 | 0.09 | 0.20 | 0.00 |
| RPL7AP34 | 0.21 | 0.05 | 0.25 | 0.25 | 0.30 | 0.21 | 0.02 |
| CTTNBP2 | 0.08 | 0.02 | 0.07 | 0.18 | 0.11 | 0.21 | 0.00 |
| LINC02585 | 4.66 | 1.65 | 3.91 | 0.35 | 0.02 | 0.22 | 0.01 |
| ARGFXP2 | 0.26 | 0.18 | 0.60 | 0.68 | 0.30 | 0.22 | 0.04 |
| TCP1P1 | 0.10 | 0.04 | 0.18 | 0.40 | 0.03 | 0.23 | 0.00 |
| RNF113B | 0.16 | 0.09 | 0.40 | 0.61 | 0.27 | 0.24 | 0.00 |
| MTHFD1P1 | 0.10 | 0.07 | 0.29 | 0.75 | 0.74 | 0.25 | 0.01 |
| TCP1P1 | 0.10 | 0.04 | 0.18 | 0.40 | 0.03 | 0.25 | 0.00 |
| RN7SL481P | 0.81 | 0.42 | 1.11 | 0.52 | 0.45 | 0.25 | 0.05 |
| RPL7AP34 | 0.21 | 0.05 | 0.25 | 0.25 | 0.30 | 0.25 | 0.02 |
| RGPD1 | 0.03 | 0.01 | 0.05 | 0.37 | 0.17 | 0.25 | 0.01 |
| FGL1 | 0.05 | 0.02 | 0.06 | 0.37 | 0.09 | 0.25 | 0.02 |
| RGPD1 | 0.03 | 0.01 | 0.05 | 0.37 | 0.17 | 0.26 | 0.01 |
| HCAR2 | 0.13 | 0.02 | 0.09 | 0.17 | 0.10 | 0.26 | 0.01 |
| GRIN2C | 0.05 | 0.04 | 0.08 | 0.81 | 0.39 | 0.28 | 0.02 |
| ARGFXP2 | 0.26 | 0.18 | 0.60 | 0.68 | 0.30 | 0.30 | 0.04 |
| RASL12 | 0.22 | 0.07 | 0.23 | 0.33 | 0.01 | 0.30 | 0.00 |
| BNIP3P1 | 0.26 | 0.18 | 0.36 | 0.71 | 0.32 | 0.30 | 0.03 |
| SERPINB5 | 0.19 | 0.12 | 0.22 | 0.65 | 0.40 | 0.31 | 0.02 |
| RASL12 | 0.22 | 0.07 | 0.23 | 0.33 | 0.01 | 0.31 | 0.00 |
| FANCD2OS | 0.08 | 0.03 | 0.11 | 0.41 | 0.24 | 0.33 | 0.03 |

Supplemental Table 4. Up- or down-regulated expressions of lipid metabolism-associated DEGs after interfering with ACSL5 after lysoPC treatment

| Gene name | ACSL5^NC^ + Vehicle Mean | ACSL5*^NC^* + lysoPC  Mean | ACSL5*^KD^* + lysoPC  Mean | ACSL5*^NC^* + lysoPC  / ACSL5*^NC^* + Vehicle  Fold change | P value | ACSL5*^NC^* + lysoPC  / ACSL5*^KD^* + lysoPC  Fold change | P value |
| --- | --- | --- | --- | --- | --- | --- | --- |
| FITM2 | 4.63 | 3.23 | 3.83 | 0.70 | 0.01 | 0.84 | 0.01 |
| ATP8A1 | 0.15 | 0.06 | 0.10 | 0.37 | 0.08 | 0.57 | 0.04 |
| TXNIP | 46.51 | 22.73 | 26.68 | 0.49 | 0.01 | 0.85 | 0.01 |
| ABCA1 | 0.68 | 0.13 | 0.23 | 0.20 | 0.03 | 0.58 | 0.03 |
| LIPH | 2.22 | 1.31 | 1.77 | 0.59 | 0.02 | 0.74 | 0.02 |
| ALDH9A1 | 40.45 | 31.38 | 36.43 | 0.78 | 0.05 | 0.86 | 0.01 |
| ELOVL3 | 8.95 | 4.43 | 5.79 | 0.49 | 0.08 | 0.76 | 0.04 |
| UIMC1 | 6.49 | 5.88 | 6.55 | 0.91 | 0.31 | 0.90 | 0.02 |
| CROT | 2.83 | 2.07 | 2.50 | 0.73 | 0.08 | 0.83 | 0.02 |
| NAAA | 6.34 | 4.56 | 5.50 | 0.72 | 0.04 | 0.83 | 0.02 |
| CDS2 | 2.97 | 3.26 | 4.35 | 1.10 | 0.27 | 0.75 | 0.01 |
| PAFAH2 | 4.83 | 5.07 | 5.77 | 1.05 | 0.17 | 0.88 | 0.05 |
| IPPK | 1.34 | 1.68 | 2.18 | 1.26 | 0.33 | 0.77 | 0.02 |
| PTGFRN | 31.85 | 35.24 | 47.22 | 1.11 | 0.53 | 0.75 | 0.00 |
| SGPL1 | 8.17 | 9.23 | 11.91 | 1.13 | 0.45 | 0.77 | 0.00 |
| PLEKHA2 | 17.94 | 18.15 | 19.70 | 1.01 | 0.87 | 0.92 | 0.00 |
| PCYOX1 | 18.52 | 19.85 | 23.20 | 1.07 | 0.64 | 0.86 | 0.04 |
| ESR1 | 0.25 | 0.25 | 0.36 | 1.01 | 0.94 | 0.70 | 0.02 |
| PIGA | 7.86 | 8.64 | 11.67 | 1.10 | 0.33 | 0.74 | 0.00 |
| ERLIN1 | 19.22 | 19.93 | 23.52 | 1.04 | 0.67 | 0.85 | 0.04 |
| PRMT6 | 11.89 | 10.57 | 12.50 | 0.89 | 0.07 | 0.85 | 0.02 |
| STX12 | 5.34 | 4.81 | 7.29 | 0.90 | 0.36 | 0.66 | 0.00 |
| UGCG | 8.74 | 6.79 | 9.25 | 0.78 | 0.11 | 0.73 | 0.00 |
| ACBD5 | 13.34 | 12.85 | 15.69 | 0.96 | 0.69 | 0.82 | 0.01 |
| LPAR5 | 2.00 | 3.20 | 2.41 | 1.60 | 0.03 | 1.33 | 0.00 |
| B3GALT5 | 0.12 | 0.25 | 0.16 | 2.12 | 0.06 | 1.53 | 0.05 |
| APOL6 | 3.61 | 5.17 | 4.84 | 1.43 | 0.03 | 1.07 | 0.01 |
| MGLL | 6.11 | 15.91 | 12.81 | 2.61 | 0.00 | 1.24 | 0.00 |
| SYNJ2 | 2.43 | 4.54 | 3.33 | 1.87 | 0.01 | 1.36 | 0.00 |
| UGGT1 | 27.02 | 37.41 | 42.15 | 1.38 | 0.08 | 0.89 | 0.02 |
| STS | 16.39 | 20.75 | 22.70 | 1.27 | 0.08 | 0.91 | 0.01 |
| STT3A | 32.12 | 39.48 | 41.13 | 1.23 | 0.05 | 0.96 | 0.03 |
| ELOVL5 | 60.58 | 70.22 | 78.69 | 1.16 | 0.00 | 0.89 | 0.02 |
| ZDHHC18 | 7.63 | 10.62 | 13.30 | 1.39 | 0.04 | 0.80 | 0.01 |
| ALG3 | 21.16 | 24.71 | 26.70 | 1.17 | 0.05 | 0.93 | 0.03 |
| PTGES | 151.38 | 223.39 | 194.93 | 1.48 | 0.04 | 1.15 | 0.01 |
| CRABP2 | 7.21 | 7.88 | 5.53 | 1.09 | 0.74 | 1.42 | 0.03 |
| ACSL5 | 1.63 | 2.47 | 0.92 | 1.51 | 0.00 | 2.69 | 0.00 |
| PRKAB1 | 4.52 | 4.87 | 3.85 | 1.08 | 0.31 | 1.27 | 0.03 |
| PHOSPHO1 | 1.29 | 1.01 | 0.49 | 0.78 | 0.22 | 2.06 | 0.01 |
| B4GALNT2 | 4.25 | 3.93 | 2.56 | 0.92 | 0.38 | 1.54 | 0.01 |
| PLA2G15 | 6.79 | 6.20 | 3.34 | 0.91 | 0.26 | 1.86 | 0.00 |
| SPTLC1 | 11.48 | 10.93 | 6.52 | 0.95 | 0.71 | 1.68 | 0.00 |
| OSBPL10 | 2.86 | 3.04 | 2.71 | 1.06 | 0.39 | 1.12 | 0.05 |
| NPC1L1 | 0.08 | 0.10 | 0.03 | 1.27 | 0.31 | 3.85 | 0.02 |
| RDH5 | 2.20 | 1.09 | 0.64 | 0.49 | 0.03 | 1.69 | 0.05 |
| ATP8B3 | 3.67 | 3.09 | 2.53 | 0.84 | 0.12 | 1.22 | 0.02 |
| DGKA | 4.80 | 4.49 | 3.64 | 0.94 | 0.48 | 1.23 | 0.01 |
| ANXA9 | 29.15 | 16.41 | 13.03 | 0.56 | 0.02 | 1.26 | 0.00 |
| CYP27A1 | 0.15 | 0.10 | 0.04 | 0.67 | 0.30 | 2.76 | 0.02 |

Supplemental Table 5. Clinical phenomes of patients for clinical studies.

|  |  | Healthy (n=290) | ADC (n=63) | SCLC (n=12) | SCC (n=21) | Acute pneumonia (n=120) | COPD (n=66) |
| --- | --- | --- | --- | --- | --- | --- | --- |
| Gender | Male (n) | 193 | 35 | 12 | 20 | 83 | 66 |
|  | Female (n) | 97 | 28 | 0 | 1 | 37 | 0 |
| Age (years) | | 42.18±11.62 | 61.11±9.91 | 60.92±7.23 | 65.29±7.57 | 57.00±14.79 | 66.98±9.22 |

Age was shown as mean ± SD

**Clinical information for patients with acute pneumonia and COPD**

|  | Acute pneumonia | | COPD | |
| --- | --- | --- | --- | --- |
| Clinical Information | Mean | SD | Mean | SD |
| Sputum | 1.80 | 1.63 | 0.74 | 0.92 |
| Chest Distress | 1.10 | 1.63 | 0.09 | 0.55 |
| Dyspnea | 1.47 | 1.70 | 1.02 | 0.92 |
| Limitation Of Motion | 0.63 | 1.16 | 0.86 | 0.76 |
| Orthopnea At Night | 0.27 | 1.01 | 0.00 | 0.00 |
| Edema Of Lower Extremity | 0.80 | 1.63 | 0.12 | 0.69 |
| Chill | 0.00 | 0.00 | 0.00 | 0.00 |
| Fever (℃) | 0.63 | 1.27 | 0.02 | 0.12 |
| Hepatojugular Reflex | 0.00 | 0.00 | 0.00 | 0.00 |
| Abdominal Distension | 0.13 | 0.51 | 0.00 | 0.00 |
| Abdominal Pain | 0.00 | 0.00 | 0.00 | 0.00 |
| Diarrhea | 0.13 | 0.73 | 0.00 | 0.00 |
| Somnolence | 0.00 | 0.00 | 0.00 | 0.00 |
| Cyanosis | 0.00 | 0.00 | 0.00 | 0.00 |
| Intracranial Hemorrhage | 0.00 | 0.00 | 0.00 | 0.00 |
| Shock | 0.00 | 0.00 | 0.00 | 0.00 |
| Syncope | 0.00 | 0.00 | 0.00 | 0.00 |
| Dysphoria | 0.13 | 0.73 | 0.00 | 0.00 |
| Nocturia Increased | 0.13 | 0.73 | 0.00 | 0.00 |
| Respiratory Failure | 0.13 | 0.73 | 0.00 | 0.00 |
| Pallor | 0.00 | 0.00 | 0.00 | 0.00 |
| ADA | 0.00 | 0.00 | 0.00 | 0.00 |
| Crackle Or Velcro | 0.00 | 0.00 | 0.00 | 0.00 |
| Pleural Thickening | 1.47 | 1.96 | 0.12 | 0.69 |
| Acropachia | 0.00 | 0.00 | 0.00 | 0.00 |
| Tired | 0.23 | 0.82 | 0.00 | 0.00 |
| Increased bronchovascular shadows | 0.53 | 1.38 | 0.48 | 1.32 |
| Muscular Soreness | 0.00 | 0.00 | 0.00 | 0.00 |
| The Thoracic Compliance Decreased | 0.27 | 1.01 | 0.00 | 0.00 |
| Mind | 0.03 | 0.18 | 0.00 | 0.00 |
| Appetite | 0.10 | 0.40 | 0.00 | 0.00 |
| Urine | 0.00 | 0.00 | 0.00 | 0.00 |
| Hoarseness | 0.07 | 0.37 | 0.00 | 0.00 |
| Superior Vena Cava Syndrome | 0.00 | 0.00 | 0.00 | 0.00 |
| Horner Syndrome | 0.00 | 0.00 | 0.00 | 0.00 |
| Brachial Plexus Compression | 0.00 | 0.00 | 0.00 | 0.00 |
| Blood Pressure | 0.83 | 1.53 | 0.17 | 0.71 |
| Diabetes | 0.53 | 1.38 | 0.61 | 1.45 |
| Family History of Lung Cancer | 0.00 | 0.00 | 0.00 | 0.00 |
| History of Asthma | 0.17 | 0.75 | 0.08 | 0.32 |
| Past History of COPD | 0.53 | 1.28 | 0.17 | 0.51 |
| Tuberculosis History | 0.40 | 1.22 | 0.15 | 0.71 |
| A History of Other Lung Diseases | 0.17 | 0.75 | 0.23 | 0.49 |
| Smoking (Pack * Years) | 0.37 | 0.85 | 0.80 | 1.39 |
| Carcinogenic Living and Occupational Environments | 0.00 | 0.00 | 1.30 | 1.87 |
| Coronary Heart Disease | 0.40 | 0.89 | 0.11 | 0.31 |
| Other chronic diseases | 0.23 | 0.77 | 0.64 | 0.94 |
| Respiratory Rate (Times /Min) | 0.43 | 0.90 | 0.00 | 0.00 |
| Nutriture | 0.83 | 0.46 | 0.03 | 0.17 |
| Lung Palpation | 0.00 | 0.00 | 0.00 | 0.00 |
| Percussion of the Lung | 0.13 | 0.73 | 0.00 | 0.00 |
| Lung Auscultation | 0.60 | 1.28 | 0.00 | 0.00 |
| Inappetence | 0.17 | 0.75 | 0.00 | 0.00 |
| Insomnia | 0.20 | 0.81 | 0.00 | 0.00 |
| Anxiety | 0.00 | 0.00 | 0.00 | 0.00 |
| Difficulty Urinating or Defecating | 0.13 | 0.73 | 0.00 | 0.00 |
| PS Scores | 0.37 | 0.85 | 0.00 | 0.00 |
| Pain (Liver Area) | 0.00 | 0.00 | 0.00 | 0.00 |
| Jaundice | 0.00 | 0.00 | 0.00 | 0.00 |
| Anemia | 0.00 | 0.00 | 0.00 | 0.00 |
| Breast Palpation | 0.00 | 0.00 | 0.00 | 0.00 |
| Chest Percussion | 0.00 | 0.00 | 0.00 | 0.00 |
| Chest Auscultation | 1.07 | 1.80 | 0.06 | 0.49 |
| Trachea Is in The Midline | 0.00 | 0.00 | 0.00 | 0.00 |
| Both Lungs Surd | 0.00 | 0.00 | 0.00 | 0.00 |
| Respiratory Sounds in Both Lungs Are Symmetrical | 0.27 | 1.01 | 0.00 | 0.00 |
| Language Fibrillation Enhanced | 0.00 | 0.00 | 0.00 | 0.00 |
| Wheezing Rale | 0.30 | 1.02 | 0.00 | 0.00 |
| Heart Visual Examination | 0.13 | 0.73 | 0.00 | 0.00 |
| Abdomen And Others | 0.00 | 0.00 | 0.00 | 0.00 |
| Osteoarthropathy Hypertrophic Pneumic | 0.00 | 0.00 | 0.00 | 0.00 |
| Gynecomastia | 0.00 | 0.00 | 0.00 | 0.00 |
| Cushing'S Syndrome | 0.00 | 0.00 | 0.00 | 0.00 |
| Syndrome Of Inappropriate Secretion of Antidiuretic Hormone | 0.00 | 0.00 | 0.00 | 0.00 |
| Neuromuscular Syndrome | 0.00 | 0.00 | 0.00 | 0.00 |
| Carcinoid Syndrome Caused by Excessive Serotonin Production | 0.00 | 0.00 | 0.00 | 0.00 |
| Multiple Peripheral Neuritis | 0.00 | 0.00 | 0.00 | 0.00 |
| Myasthenia Syndrome | 0.00 | 0.00 | 0.00 | 0.00 |
| TBil | 0.00 | 0.00 | 0.00 | 0.00 |
| Albumin（G/L) | 0.17 | 0.46 | 0.00 | 0.00 |
| Urea | 0.27 | 0.87 | 0.03 | 0.25 |
| Creatinine | 0.03 | 0.18 | 0.00 | 0.00 |
| Total Cholesterol | 0.00 | 0.00 | 0.05 | 0.27 |
| Triglyceride | 0.00 | 0.00 | 0.00 | 0.00 |
| HDL | 0.13 | 0.73 | 0.02 | 0.12 |
| LDL | 0.00 | 0.00 | 0.00 | 0.00 |
| Na (mmol/L) | 0.07 | 0.37 | 0.00 | 0.00 |
| K(mmol/L) | 0.23 | 0.43 | 0.00 | 0.00 |
| Ca (mmol/L) | 0.40 | 1.22 | 0.00 | 0.00 |
| P (mmol/L） | 0.40 | 1.22 | 0.00 | 0.00 |
| Mg (mmol/L) | 0.00 | 0.00 | 0.00 | 0.00 |
| Glycosylated Hemoglobin | 0.00 | 0.00 | 0.00 | 0.00 |
| Sao2(%) | 0.17 | 0.46 | 0.20 | 0.40 |
| CRP | 1.13 | 1.36 | 0.02 | 0.12 |
| Blood glucose (mmol/L) | 0.90 | 1.47 | 0.50 | 1.17 |
| Hemoglobin(G/L) | 0.20 | 0.48 | 0.08 | 0.51 |
| Leukocyte | 1.20 | 1.86 | 0.24 | 0.96 |
| Neutrophile Granulocyte | 0.67 | 1.52 | 0.00 | 0.00 |
| ALT | 0.07 | 0.37 | 0.02 | 0.12 |
| AST | 0.03 | 0.18 | 0.00 | 0.00 |
| Alkaline Phosphatase | 0.00 | 0.00 | 0.00 | 0.00 |
| γ一GT | 0.40 | 0.93 | 0.00 | 0.00 |
| D- dimer | 2.00 | 2.03 | 0.12 | 0.69 |
| Trioxypurine(μmol/L) | 0.00 | 0.00 | 0.00 | 0.00 |
| PH | 0.27 | 1.01 | 0.00 | 0.00 |
| Pao_2_(mmHg) | 0.67 | 1.12 | 0.06 | 0.49 |
| Paco_2_(mmHg) | 0.43 | 1.22 | 0.06 | 0.49 |
| SCC | 0.00 | 0.00 | 0.00 | 0.00 |
| CEA | 0.03 | 0.18 | 0.00 | 0.00 |
| Cyfra211 | 0.00 | 0.00 | 0.00 | 0.00 |
| Emphysema | 0.80 | 1.63 | 0.85 | 1.65 |

**Clinical information of lung cancer patients**

|  | ADC | | SCC | | SCLC | |
| --- | --- | --- | --- | --- | --- | --- |
| Clinical Information | Mean | SD | Mean | SD | Mean | SD |
| Cough | 0.76 | 0.93 | 1.62 | 0.74 | 1.17 | 1.03 |
| Sputum | 0.46 | 0.90 | 1.29 | 0.85 | 0.42 | 0.79 |
| Haemoptysis | 0.08 | 0.28 | 0.48 | 0.60 | 0.25 | 0.45 |
| Dyspnea | 0.22 | 0.48 | 0.29 | 0.96 | 0.42 | 1.16 |
| Hoarse Sound | 0.05 | 0.33 | 0.10 | 0.44 | 0.17 | 0.58 |
| Fatigue | 0.19 | 0.70 | 0.00 | 0.00 | 0.00 | 0.00 |
| Appetite | 0.51 | 1.10 | 0.38 | 0.80 | 0.33 | 0.78 |
| Sleep | 0.22 | 0.63 | 0.00 | 0.00 | 0.00 | 0.00 |
| Anxiety | 0.03 | 0.16 | 0.00 | 0.00 | 0.00 | 0.00 |
| Weight Loss (Compare with Former) | 0.49 | 1.12 | 0.48 | 1.25 | 0.17 | 0.58 |
| PS Score | 0.03 | 0.16 | 0.14 | 0.48 | 0.00 | 0.00 |
| Mind | 0.00 | 0.00 | 0.00 | 0.00 | 0.00 | 0.00 |
| Fever | 0.03 | 0.16 | 0.19 | 0.51 | 0.17 | 0.39 |
| Heart Rate | 0.11 | 0.66 | 0.00 | 0.00 | 0.00 | 0.00 |
| Respiratory Rate | 0.11 | 0.66 | 0.00 | 0.00 | 0.00 | 0.00 |
| Blood Pressure(mmHg) | 0.14 | 0.42 | 0.05 | 0.22 | 0.42 | 1.16 |
| Chest Palpation | 0.00 | 0.00 | 0.00 | 0.00 | 0.00 | 0.00 |
| Chest Percussion | 0.32 | 1.11 | 0.24 | 0.89 | 0.00 | 0.00 |
| Heart | 0.00 | 0.00 | 0.00 | 0.00 | 0.00 | 0.00 |
| Lung Cancer Family History | 0.03 | 0.16 | 0.00 | 0.00 | 0.00 | 0.00 |
| COPD History | 0.03 | 0.16 | 0.14 | 0.36 | 0.25 | 0.62 |
| Other Lung Disease History | 0.43 | 1.07 | 0.00 | 0.00 | 0.33 | 1.15 |
| Smoking (Packet*Year) | 0.59 | 1.09 | 1.43 | 1.43 | 1.25 | 1.76 |
| Cancerigenic Occupational Environment | 0.00 | 0.00 | 0.00 | 0.00 | 0.00 | 0.00 |
| T(Tumor) | 1.62 | 1.71 | 1.71 | 1.65 | 3.33 | 1.23 |
| Sum Of All T (mm) | 1.05 | 1.03 | 0.95 | 1.24 | 1.50 | 0.90 |
| N (Lymph Node) | 2.22 | 1.46 | 1.86 | 1.53 | 3.08 | 1.44 |
| Metastasis (Brain) | 0.73 | 1.43 | 0.29 | 0.90 | 0.08 | 0.29 |
| Metastasis (Bone) | 0.78 | 1.47 | 0.52 | 1.21 | 0.92 | 1.16 |
| Metastasis (Liver) | 0.08 | 0.36 | 0.05 | 0.22 | 0.00 | 0.00 |
| Metastasis (Adrenal Gland) | 0.05 | 0.23 | 0.10 | 0.44 | 0.17 | 0.39 |
| Metastasis (Other) | 0.38 | 1.11 | 0.05 | 0.22 | 0.00 | 0.00 |
| Onset Of New Lesion (Metastasis) | 1.08 | 1.80 | 0.00 | 0.00 | 0.33 | 1.15 |
| ALT | 0.32 | 0.94 | 0.05 | 0.22 | 0.08 | 0.29 |
| AST | 0.16 | 0.73 | 0.05 | 0.22 | 0.08 | 0.29 |
| Urea (mmol/L) | 0.05 | 0.33 | 0.05 | 0.22 | 0.00 | 0.00 |
| Creatinine (μmol/L) | 0.00 | 0.00 | 0.00 | 0.00 | 0.00 | 0.00 |
| Uric Acid | 0.30 | 1.02 | 0.00 | 0.00 | 0.00 | 0.00 |
| Na(mmol/L) | 0.11 | 0.31 | 0.05 | 0.22 | 0.17 | 0.39 |
| K(mmol/L) | 0.05 | 0.23 | 0.05 | 0.22 | 0.08 | 0.29 |
| Cl (mmol/L) | 0.22 | 0.92 | 0.19 | 0.87 | 0.33 | 1.15 |
| Ca(mmol/L) | 0.11 | 0.39 | 0.10 | 0.30 | 0.00 | 0.00 |
| P (mmol/L） | 0.22 | 0.92 | 0.19 | 0.87 | 0.00 | 0.00 |
| PH | 0.00 | 0.00 | 0.00 | 0.00 | 0.00 | 0.00 |
| PaO_2_(mmHg) | 0.35 | 0.59 | 0.29 | 0.46 | 0.25 | 0.45 |
| PaCO_2_(mmHg) | 0.00 | 0.00 | 0.00 | 0.00 | 0.00 | 0.00 |
| SCC | 0.30 | 0.97 | 0.00 | 0.00 | 0.00 | 0.00 |
| Cyfra211 | 0.27 | 0.51 | 1.14 | 1.56 | 0.25 | 0.62 |
| NSE | 0.14 | 0.35 | 0.14 | 0.36 | 0.92 | 1.16 |
| CRP | 1.22 | 1.70 | 1.38 | 1.86 | 1.75 | 1.82 |
| PT(s) | 0.11 | 0.66 | 0.29 | 0.90 | 0.00 | 0.00 |
| Free Blood Glucose (mmol/L) | 0.22 | 0.58 | 0.19 | 0.40 | 0.17 | 0.58 |
| CEA | 0.65 | 1.40 | 0.43 | 1.21 | 0.00 | 0.00 |

Supplemental Table 6. Seven types of airway and lung epithelial cells were characterized and labeled with corresponding cell-specific genes.

| Names of epithelia | Marker genes | Healthy | COPD | IPF | ADC | Para-cancer | SSC |
| --- | --- | --- | --- | --- | --- | --- | --- |
| Alveolar epithelial type I | HOPX | 10.22±12.42 | 5.55±8.69 | 7.21±10.4 | 7.89±8.72 | 5.69±6.31 | 14.64±14.54 |
|  | PDPN | 0.29±0.69 | 0.47±0.84 | 0.27±0.59 | 0.36±0.65 | 0.44±0.79 | 0.38±0.86 |
|  | AGER | 9.83±9.56 | 11.08±11.52 | 8.58±10.29 | 20.89±29.17 | 20.47±21.81 | 16.98±18.53 |
|  | CLDN18 | 2.19±2.98 | 2.74±3.16 | 4.6±5.23 | 3.61±3.62 | 2.62±3.13 | 4.7±7.92 |
|  | EMP2 | 14.18±13.85 | 19.02±13.88 | 18.1±16.33 | 20.3±21.9 | 20.26±16.52 | 28.64±33.29 |
| Alveolar epithelial type II | SFTPB | 111.55±88.39 | 70.12±77.8 | 91.38±108.84 | 89.06±92.62 | 108.27±61.19 | 133.16±98.09 |
|  | SFTPC | 744.74±688.36 | 308.11±405.66 | 306.45±458.55 | 525.69±777.57 | 1519.68±950.35 | 1033.47±1094.82 |
|  | SFTPD | 16.73±17.75 | 6.72±8.52 | 5.43±7 | 15.02±27.61 | 23.45±17.5 | 14.99±15.21 |
|  | ETV5 | 0.88±1.25 | 0.95±2.03 | 0.81±1.71 | 0.4±0.81 | 0.94±1.22 | 0.76±1.33 |
|  | MUC1 | 1.52±3.28 | 0.03±0.2 | 0.1±0.51 | 12.95±13.47 | 9.15±6.72 | 12.07±10.04 |
|  | WIF1 | 3.17±3.55 | 1.53±2.29 | 1.93±3.22 | 0.68±1.86 | 4.06±4.61 | 4.2±5.28 |
|  | HHIP | 2.39±3.27 | 0.39±1.05 | 0.43±0.98 | 0.95±2.48 | 2.41±3.07 | 2.25±3.94 |
| Basal epithelia | KRT14 | 0.08±0.46 | 0.07±0.39 | 0.28±1.59 | 0.36±1.16 | 0±0 | 1.07±7 |
|  | KRT5 | 1.92±3.81 | 1.18±2.54 | 2.78±7.95 | 1.36±2.9 | 1.17±1.37 | 6.94±12.29 |
| Ciliated epithelia | FOXJ1 | 2.55±3.73 | 2.1±3.23 | 2.05±3.13 | 4.62±6.22 | 3.61±4.52 | 4.03±4.51 |
| Club epithelia | SCGB1A1 | 285.87±671.8 | 386.85±921.35 | 144.4±481.59 | 14.05±201.05 | 842.83±1360.31 | 136.31±606.16 |
|  | SCGB3A2 | 186.35±318.24 | 144.13±259.77 | 110.63±221.43 | 67.28±195.4 | 284.01±404.37 | 88.66±195.79 |
| Goblet epithelia | MUC5AC | 17.46±20.91 | 1.2±2.2 | 7.05±19.79 | 21.5±30.41 | 1±1 | 7.23±13.68 |
|  | MUC5B | 0.54±1.2 | 5.3±9.38 | 2.92±4.84 | 30±36.77 | 9±7.81 | 4.38±6.64 |
| Mucous epithelia | MUC5AC | 3.94±10.5 | 0.11±0.65 | 0.35±1.92 | 0.35±0.81 | 0.92±3.56 | 0.75±3.22 |

Supplemental Table 7. The pathological score criteria

| Score | Description |
| --- | --- |
| Inflammatory infiltrates | |
| 0 | no obvious inflammatory cell infiltration |
| 1 | small amount of inflammatory cells infiltrate |
| 2 | More inflammatory cell infiltration |
| 3 | A large number of inflammatory cells infiltrate |
| Hemorrhage | |
| 0 | no significant hemorrhage or congestion |
| 1 | Small area of vascular congestion |
| 2 | Larger vascular congestion or mild small bleeding |
| 3 | Large range of vascular congestion or bleeding |
| 4 | Massive bleeding |
| Thickened alveolar wall (for lung only) | |
| 0 | The morphology of alveoli was intact, no signs of thickened alveolar wall |
| 1 | The alveolar wall was slightly thickened |
| 2 | The alveolar wall was moderately thickened and the alveolar cavity was narrowed |
| 3 | The alveolar wall thickened severely and the alveolar cavity narrowed to disappear |

The final score is the total score of three items, the score range is 0-10 points.
