# Supplementary figures and images for: Lysophosphatidylcholine inhibits lung cancer cell proliferation by regulating fatty acid metabolism enzyme long‐chain acyl‐coenzyme A synthase 5
Source: Clin Transl Med. 2023 Jan 13;13(1):e1180. doi: 10.1002/ctm2.1180 (PMC9839868; doi:10.1002/ctm2.1180)

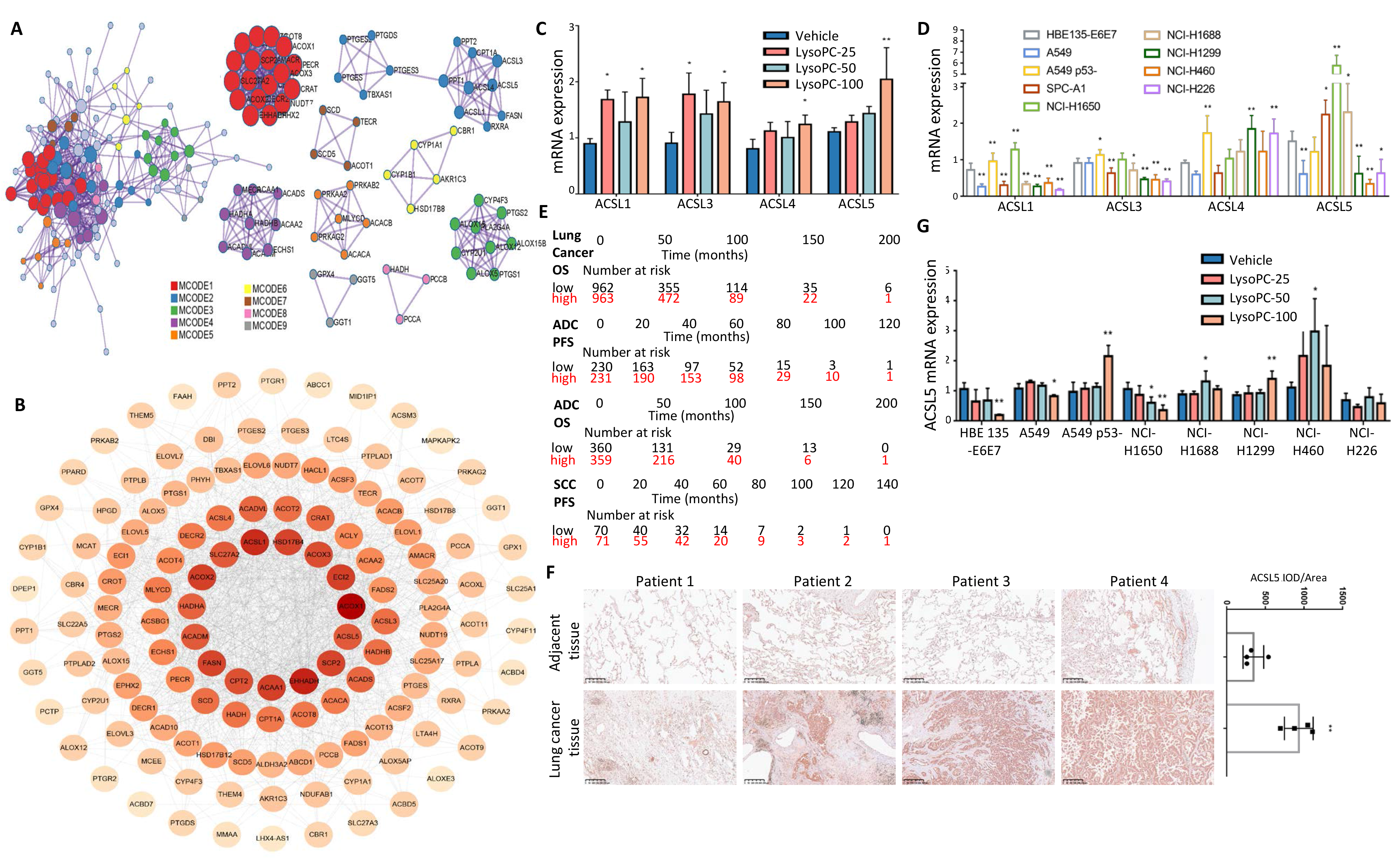

Supplement: Supplementary file 2 — Supporting Information [file CTM2-13-e1180-s005.tif]

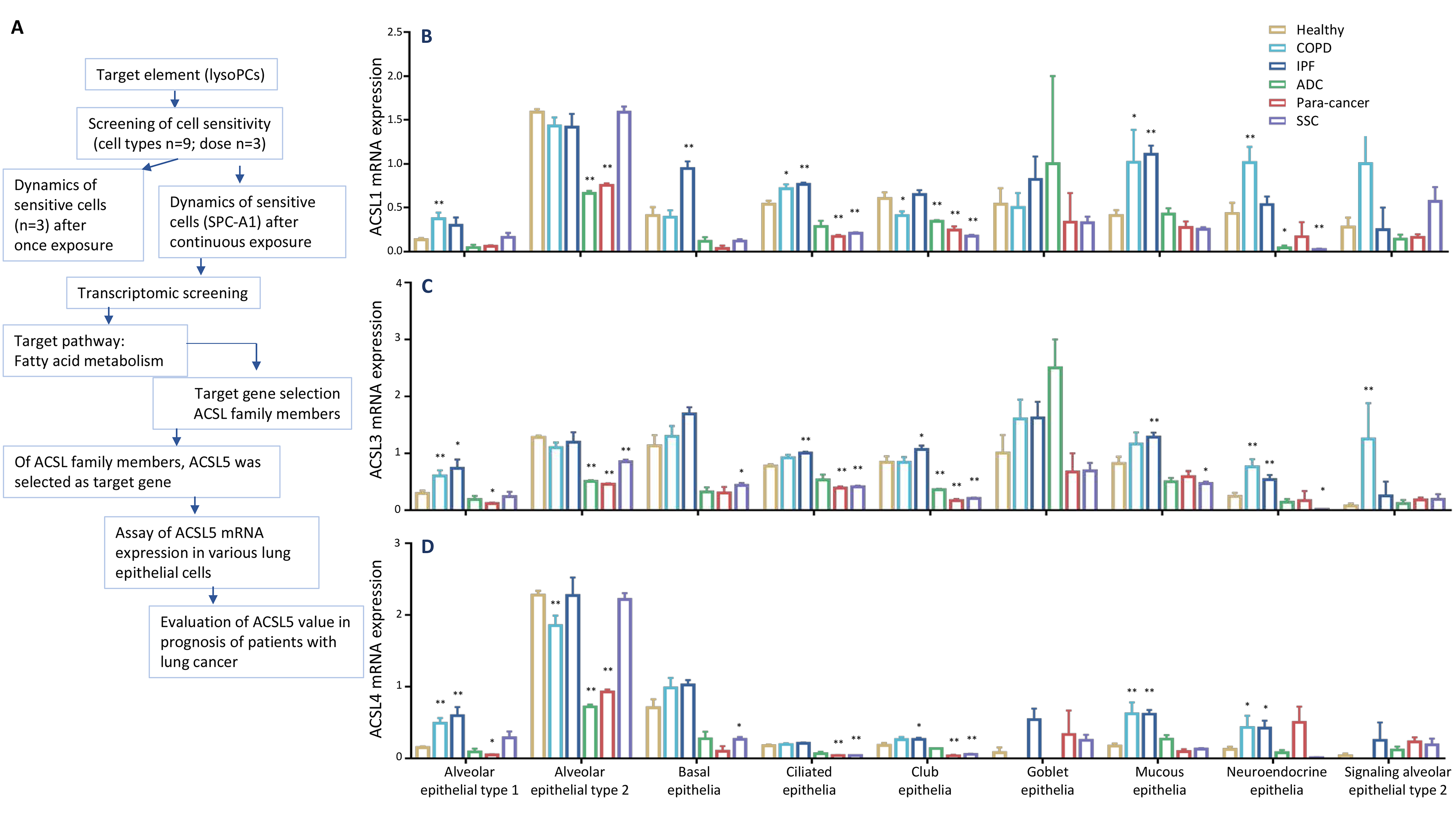

Supplement: Supplementary file 3 — Supporting Information [file CTM2-13-e1180-s008.tif]

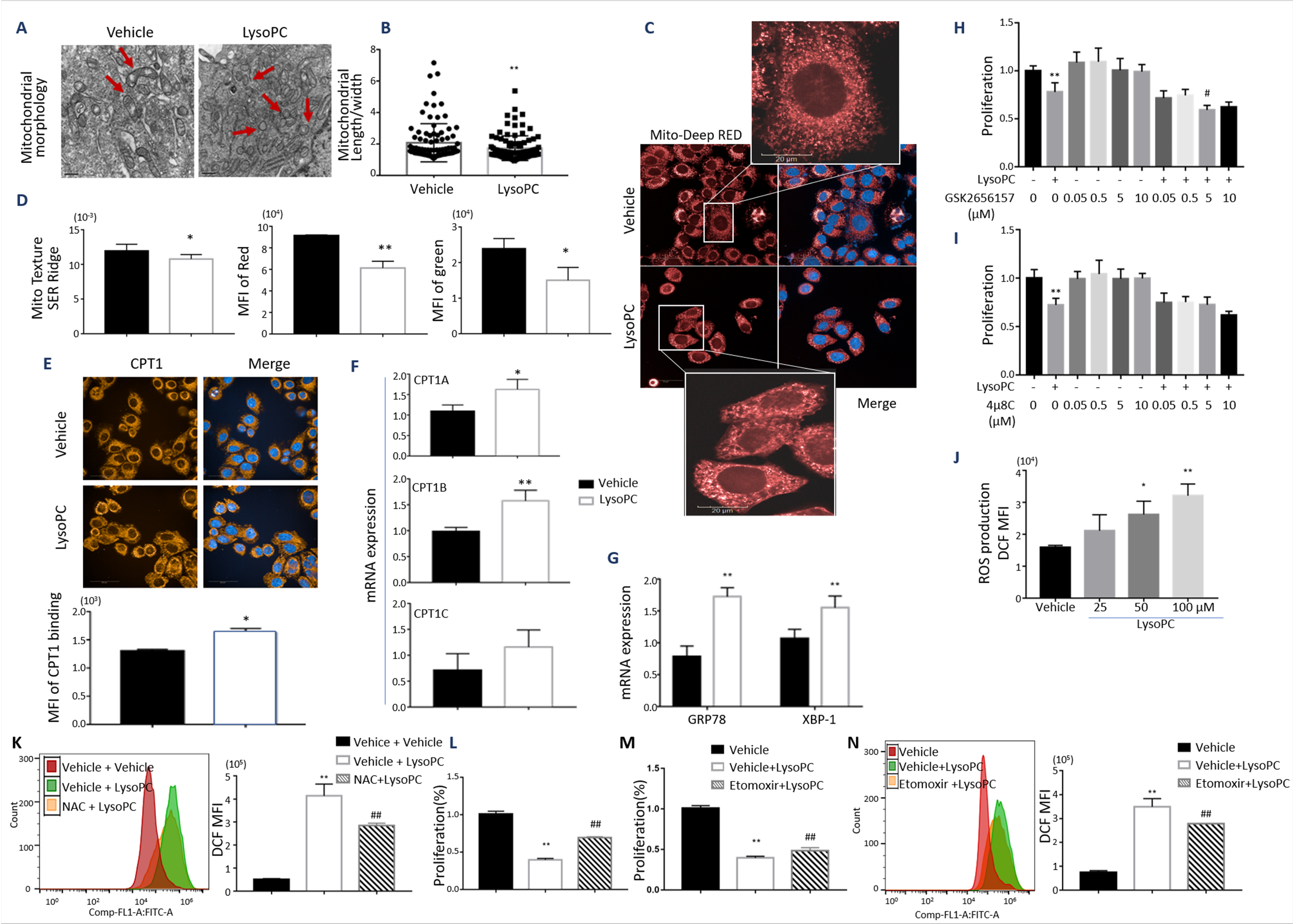

Supplement: Supplementary file 4 — Supporting Information [file CTM2-13-e1180-s001.tif]

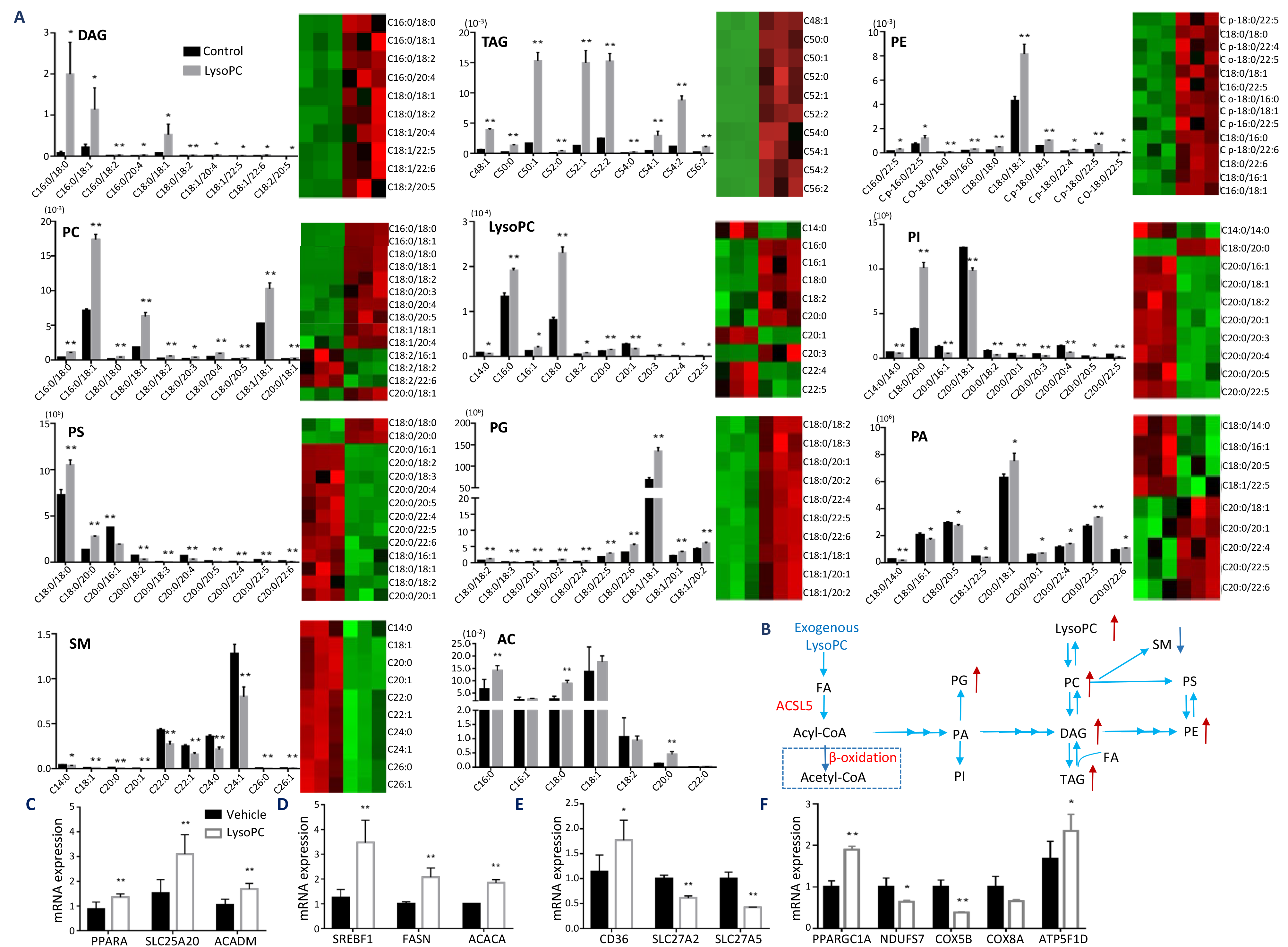

Supplement: Supplementary file 5 — Supporting Information [file CTM2-13-e1180-s007.tif]

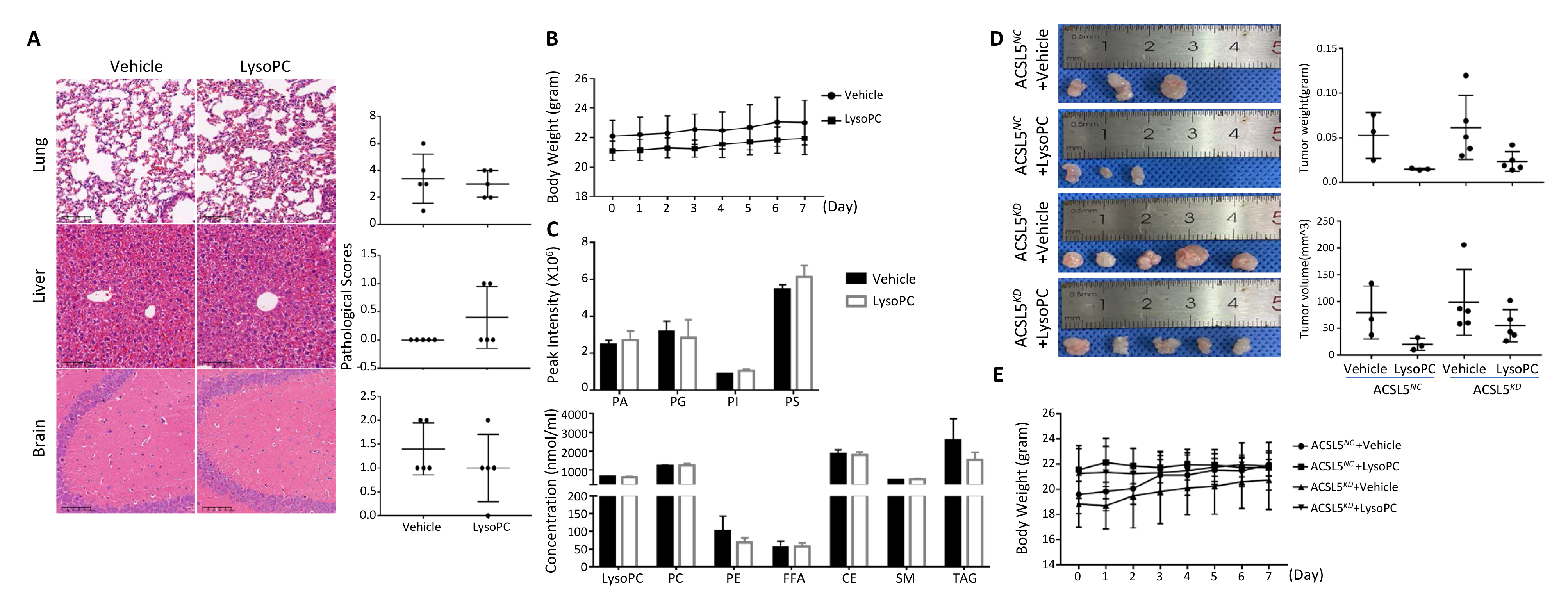

Supplement: Supplementary file 6 — Supporting Information [file CTM2-13-e1180-s006.tif]

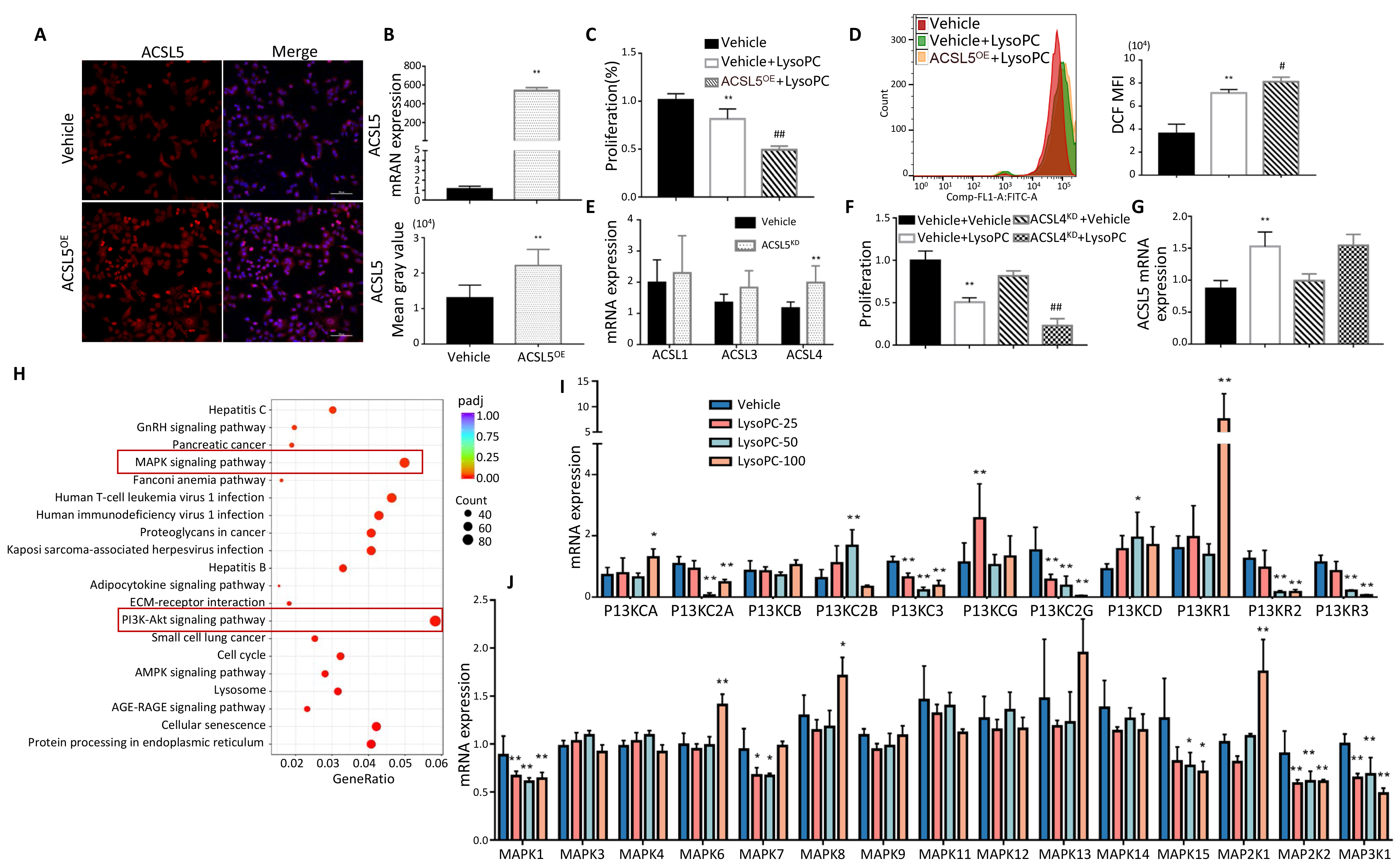

Supplement: Supplementary file 7 — Supporting Information [file CTM2-13-e1180-s004.tif]

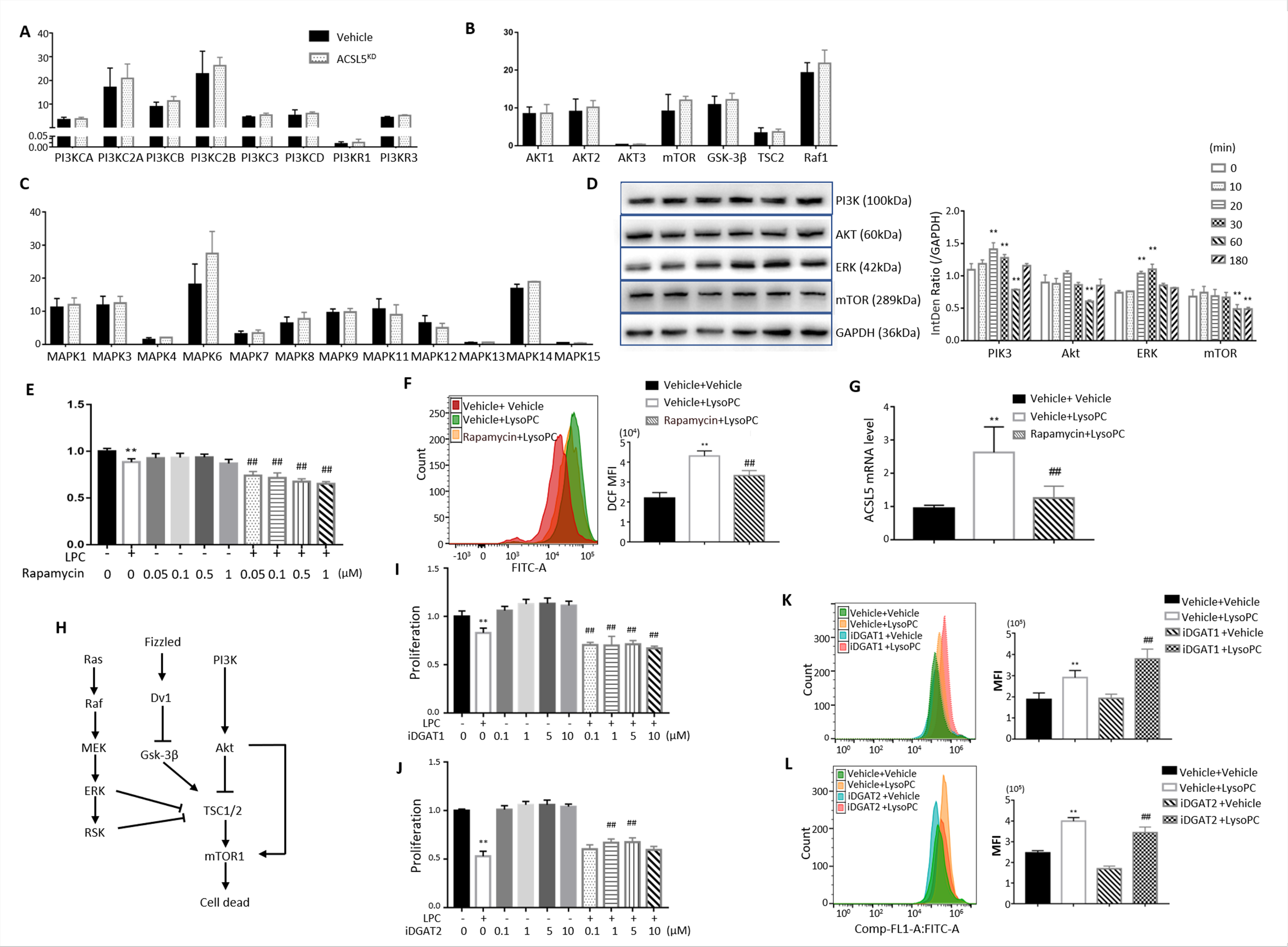

Supplement: Supplementary file 8 — Supporting Information [file CTM2-13-e1180-s003.tif]
